# Design and Physicochemical Characterization of a Multifunctional Maisine-Based Microemulsion Incorporating Doxorubicin@Mn-Doped Magnetite Nanoparticles for MRI, Hyperthermia, and Drug Delivery

**DOI:** 10.3390/nano16171065

**Published:** 2026-08-26

**Authors:** Mirela Nistor, Daniel Gherca, Cristina Mariana Uritu, Marian Grigoras, Vera Balan, Raluca M. Fratila, Rares-Ionut Stiufiuc, Brindusa Dragoi, Aurel Pui

**Affiliations:** 1 Nanotechnology Laboratory, TRANSCEND Department, Regional Institute of Oncology, 2-4 General Henri Mathias Berthelot, 700483 Iasi, Romania; mirela.nistor@chem.uaic.ro (M.N.); balan.vera@umfiasi.ro (V.B.); rares.stiufiuc@umfcluj.ro (R.-I.S.); 2 Faculty of Chemistry, “Alexandru Ioan Cuza” University of Iasi, 11 Carol I Blvd., 700506 Iasi, Romania; 3 National Institute of Research and Development for Technical Physics, 700050 Iasi, Romania; dgherca@phys-iasi.ro (D.G.); mgrigoras@phys-iasi.ro (M.G.); 4 Advanced Centre for Research-Development in Experimental Medicine “Prof. Ostin C. Mungiu” (CEMEX), Grigore T. Popa University of Medicine and Pharmacy, 9-13 Mihail Kogalniceanu, 700259 Iasi, Romania; cristina-mariana.uritu@umfiasi.ro; 5 Biomedical Sciences Department, Faculty of Medical Bioengineering, Grigore T. Popa University of Medicine and Pharmacy of Iasi, 9-13 Kogalniceanu Street, 700454 Iasi, Romania; 6 Instituto de Nanociencia y Materiales de Aragón, INMA (CSIC-Universidad de Zaragoza), C/Pedro Cerbuna 12, 50009 Zaragoza, Spain; rfratila@unizar.es; 7 Centro de Investigación Biomédica en Red de Bioingeniería, Biomateriales y Nanomedicina (CIBER-BBN), 50018 Zaragoza, Spain; 8 Department of NanoSciences, Institute of Biomedical Research—MedFUTURE, “Iuliu Hatieganu” University of Medicine and Pharmacy, Louis Pasteur 4-6, 400349 Cluj Napoca, Romania

**Keywords:** Mn-doped magnetite nanoparticles, microemulsion, MRI contrast agents, drug release

## Abstract

Multifunctional nanocarriers capable of integrating imaging, magnetic functionality, and controlled drug delivery represent an important research topic in cancer nanomedicine. Herein, Mn-doped (Fe_3_O_4_) magnetite nanoparticles (MNPs) were engineered and incorporated into a Maisine CC-based oil-in-water microemulsion (ME) to obtain a multifunctional nanoplatform for magnetic resonance imaging (MRI), hyperthermia, and controlled drug release. A series of Mn-doped MNPs (1–10% Mn:Fe_3_O_4_) was synthesized by coprecipitation. X-ray diffraction confirmed the preservation of the cubic spinel upon Mn incorporation. The Mn incorporation resulted in MNPs made of crystallites (~9–12 nm) whose magnetic properties were improved. Also, 10% Mn led to a very good magnetic heating efficiency under alternating magnetic fields with a specific absorption rate of ~111 W·g^−1^. The obtained MNPs proved to be *T*_2_-weighted MRI contrast agents, with an increase in the *r*_2_ values up to ~844 mM^−1^·s^−1^ after incorporation into ME. The optimized composition of Mn_10%_ was subsequently loaded with doxorubicin (DOX) and integrated into ME. Drug-release studies revealed a biphasic profile, characterized by an initial burst phase followed by sustained release up to 48 h. These findings demonstrate that dopant-engineered MNPs combined with a ME carrier can provide a versatile platform for integrating magnetic hyperthermia potential, *T*_2_-weighted MRI contrast enhancement, and controlled chemotherapeutic delivery within a single nanostructured system.

## 1. Introduction

Microemulsions (MEs) have emerged as versatile nanoscale delivery platforms for therapeutic agents owing to their thermodynamic stability, ease of preparation, and capacity to solubilize both hydrophilic and hydrophobic compounds. These attributes have made MEs attractive for oncology applications, particularly for co-delivery strategies where multiple drugs must be co-encapsulated and released in a controlled manner. These properties have raised strong interest in MEs as versatile pharmaceutical carriers capable of improving drug solubility, modulating release kinetics, and enhancing bioavailability [1,2,3,4].

The development of nanocarrier-based delivery systems in oncology is driven by the need to enhance the therapeutic index of cytotoxic agents by improving tumor localization while minimizing systemic toxicity. Among clinically established chemotherapeutics, doxorubicin (DOX), an anthracycline, remains a key agent for the treatment of breast cancer, lymphomas, sarcomas, and multiple other solid, as well as hematologic malignancies. Nevertheless, the clinical application of DOX is significantly constrained by cumulative cardiotoxicity, non-specific tissue distribution, and dose-dependent systemic adverse effects. These limitations have intensified efforts in current cancer nanomedicine to engineer carrier platforms capable of modulating pharmacokinetics, enhancing tumor-selective accumulation, minimizing off-target exposure, and enabling multimodal therapeutic strategies that integrate drug delivery with imaging or externally triggered responsiveness [5,6,7].

These limitations have consequently intensified ongoing efforts in cancer nanomedicine to develop advanced carrier platforms capable of precisely modulating pharmacokinetic profiles, enhancing tumor-specific accumulation, reducing off-target exposure, and facilitating multimodal therapeutic approaches that combine drug delivery with diagnostic imaging or externally triggered responsiveness.

Such multifunctional platforms are particularly attractive in cancer treatment because they allow for simultaneous imaging and therapy, enabling tumor visualization alongside localized therapeutic intervention and monitoring of treatment response. Among the materials investigated for this purpose, magnetic nanoparticles (MNPs), particularly those that are iron oxide-based, have been extensively studied due to their superparamagnetic behavior, chemical stability, and established biocompatibility. Their response to external magnetic field makes them suitable for applications like magnetic targeting, magnetic hyperthermia (HT), and magnetic resonance imaging (MRI) [8,9,10,11].

Magnetic HT has attracted particular interest as a localized cancer treatment strategy in which MNPs generate heat when exposed to alternating current (AC) magnetic fields. The heating efficiency, commonly expressed as the specific absorption rate (SAR), depends not only on the intrinsic magnetic properties of the nanoparticles but also on their interactions and spatial organization within the surrounding environment and AMF parameters [12,13,14]. In superparamagnetic iron oxide nanoparticles (SPIONs), magnetic heating originates from field-induced magnetic losses governed by Néel and Brownian relaxation mechanisms, which are strongly influenced by intrinsic parameters, such as saturation magnetization, effective magnetic anisotropy, and hydrodynamic size. These factors collectively determine SAR under AC magnetic fields [15,16]. In parallel, iron oxide nanoparticles are widely investigated as MRI contrast agents, where their magnetic properties influence the relaxation of nearby water protons and therefore the imaging signal [17,18]. Both heating performance and MRI contrast are sensitive to nanoparticle arrangement, aggregation state, and confinement within carrier systems [10,19,20].

Beyond their imaging and heating capabilities, MNPs are also being increasingly explored as drug-delivery components in multifunctional nanocarrier systems. Their surfaces can adsorb or conjugate therapeutic molecules, while magnetic responsiveness allows for external-field guidance and potential spatial control of drug accumulation at target sites. Importantly, when MNPs are incorporated into carrier matrices, such as emulsions or lipid systems, the surrounding microstructure can influence both magnetic behavior and drug-release kinetics by modifying nanoparticle interactions, molecular diffusion, and interfacial transport processes [8,10,20]. Consequently, embedding MNPs within structured delivery systems offers the possibility to combine imaging capability, magnetically induced heating, and controlled drug release within a single therapeutic platform.

Despite the increasing interest in the development of multifunctional lipid-based nanocarriers for cancer theranostics, most of the reported nanoconstructs are based on magnetoliposomes or thermosensitive liposomes. They combined chemotherapeutic agents with magnetic HT and/or MRI within a single carrier. Recent reviews further highlight magnetoliposomes as one of the most mature classes of lipid-based magnetic nanocarriers for theranostic applications, while thermosensitive liposomes remain the predominant platform for combining chemotherapy with externally triggered release and MRI guidance [21,22,23,24]. Yet, to the best of our knowledge, ME-based theranostic nanosystems combining HT, MRI, and controlled chemotherapeutic delivery within a single formulation are not reported. Consequently, the development of such multifunctional nanoformulations represents an underexplored strategy that may broaden the application of lipid-based nanocarriers for cancer theranostics.

In this present study, we build upon our previously optimized oil-in-water ME platform [25] to develop multifunctional ME systems incorporating Mn-doped MNPs (1–10% Mn:Fe_3_O_4_) as the magnetic component, thereby enhancing their functional versatility. Specifically, we report on the design and synthesis of a multifunctional nanoformulation with potential theranostic applications that are validated at this stage of development by physicochemical characterization, MRI relaxivity measurements, HT performance, and drug-release evaluation. Among the previously investigated ME formulations, the sample denoted as F9 exhibited superior physicochemical stability and structural robustness and was selected as the reference ME matrix. To introduce magnetic functionality, Mn:Fe_3_O_4_ was synthesized, employing controlled cation doping to tune magnetic response and heat dissipation efficiency while preserving the spinel structure. It is known that the controlled introduction of Mn (1–10%) during synthesis influences cation distribution within a spinel lattice, leading to modifications in magnetic anisotropy and effective magnetic moment, which can improve heat dissipation efficiency as compared to undoped magnetite (Fe_3_O_4_) [26,27,28]. Following systematic composition screening, the selected Mn-doped formulation was integrated into the ME matrix both in its unloaded form and after adsorption of DOX, enabling direct comparison between nanoparticle-only, ME-only, and the fully integrated system.

In addition to its effects on magnetic heating, Mn incorporation introduces paramagnetic contributions that can influence proton relaxation, with Mn-containing spinel ferrites exhibiting increased transverse relaxivity and, in some cases, composition-dependent *T*_1_/*T*_2_ contrast behavior [29,30]. This approach enables the systematic investigation of the effects of magnetically responsive nanoparticle incorporation on the physicochemical properties, magnetic behavior, and drug-delivery performance of ME carriers. Therefore, this work goes beyond the conventional application of lipid MEs for drug delivery and focuses on the development of a multifunctional ME-based nanoconstruct loaded with Mn-doped MNPs and DOX. The aim is to combine magnetic HT, MRI relaxivity, and controlled drug delivery within a single nanoformulation, with particular emphasis on (i) compositional tuning of the magnetite core, (ii) magnetic heating performance under alternating magnetic fields relevant for HT, (iii) MRI-related relaxometric behavior within the confined ME environment, and (iv) controlled release of the chemotherapeutic agent. Hence, this work aims to provide a rational framework for the development of multifunctional ME-based nanocarriers for advanced cancer therapy.

## 2. Materials and Methods

### 2.1. Chemicals

Anhydrous FeCl_3_ (Sigma-Aldrich, Darmstadt, Germany), anhydrous FeCl_2_ (Sigma-Aldrich, St. Louis, MO, USA), MnCl_2_·4H_2_O (VWR, ≥99.2%, Leuven, Belgium), Tween 20 (Sigma-Aldrich, St. Louis, MO, USA), Maisine^®^ CC (Gattefossé, Saint-Priest, France) (kind gift from Azelis, Bucharest, Romania), phosphate buffer (PB, K_2_HPO_4_ Sigma-Aldrich > 99%, Darmstadt, Germany; KH_2_PO_4_ Lach:ner > 99%, Neratovice, Czech Republic), ethanol (EtOH, Honeywell, 99,8%, Seelze, Germany), Pluronic F127 (C_3_H_6_O·C_2_H_4_O)_x_ (Sigma-Aldrich, BioReagent, suitable for cell culture, St. Louis, MO, USA), NaOH (VWR Chemicals, 99.1%, Leuven, Belgium), and Doxorubicin hydrochloride (European Pharmacopoeia (EP) Reference Standard, Strasbourg, France) as a model anticancer drug were used without further purification. Distilled water was produced using a PD 4 water distillation unit (LAUDA Dr. R. Wobser GmbH & Co. KG, Burgwedel, Germany).

### 2.2. Synthesis of Mn-Doped Magnetic Nanoparticles and Doxorubicin Loading

A series of Mn-doped MNPs were synthesized using our previously established coprecipitation protocol [25]. Mn was introduced at nominal concentrations of 1%, 5%, and 10% relative to the total number of iron moles, obtaining the following samples: Mn_1%_:Fe_3_O_4_, Mn_5%_:Fe_3_O_4_, and Mn_10%_:Fe_3_O_4_, respectively. The metal precursors were dissolved in water containing 2% Pluronic F-127, which was used as a stabilizing agent to ensure colloidal stability and biocompatibility. Each reaction mixture was heated to 65 °C and stirred at 350 rpm under a nitrogen atmosphere using an Atlas HD Automated Jacketed Reactor (Atlas Software version 1.8.1). A 10% NaOH solution was added dropwise using an automated syringe pump until the reaction mixture reached pH 14. All the synthesis parameters were tuned by the accompanying control software to ensure reproducible reaction conditions. The resulting suspensions of nanoparticles were repeatedly washed with distilled water to remove unreacted precursors and excess surfactant. The final colloidal suspensions were adjusted to neutral pH and stored at 5 °C until further use.

DOX loading onto MNPs was performed with passive adsorption, a mild and straightforward approach that does not require harsh chemical conditions or activating agents, thereby preserving the physicochemical integrity of both MNPs and DOX. A calculated amount of DOX was added to an aqueous suspension of MNPs obtained immediately after synthesis, in an amount corresponding to a theoretical drug loading of 15%.

The resulting suspension was subjected to gentle stirring (100 rpm) for 24 h at room temperature under light-protected conditions to prevent DOX degradation. After 24 h, the suspension was magnetically decanted to separate DOX-loaded MNPs (DOX@MNP) from the non-adsorbed drug present in the supernatant. The supernatant was subsequently analyzed using UV–Vis spectrophotometry (λ = 480 nm) to quantify the amount of non-adsorbed DOX. Based on these data, the encapsulation efficiency (EE%) and drug loading capacity (DLC%) were calculated using the following equations:

$$EE\%=\;\frac{m_{DOX\;adsorbed}}{m_{DOX\;initial}}\;\times 100$$

$$DLC\%=\frac{m_{DOX\;adsorbed}}{m_{MNPs}+m_{DOX\;adsorbed}}\times 100$$

### 2.3. Formulation of Microemulsions with and Without MNPs

The ME system employed in the present study was based on F9 formulation, previously developed and optimized by our group [25]. This formulation was selected from a series of ten formulations due to its superior physicochemical stability over time and ability to maintain a clear, isotropic oil-in-water ME structure. F9 formulation consists of 2 g oil phase, 8 g surfactant/co-surfactant mixture (S_mix_), and 2 g aqueous phase, as reported in Table 1 of the referenced work. The same preparation protocol and processing conditions described for F9 were consistently applied to all formulations investigated in the present study to ensure methodological uniformity and enable direct comparison of their physicochemical and functional properties.

### 2.4. Physicochemical Characterization of Magnetic Nanoparticles and Microemulsions

Structural and crystallographic characterization were performed by *X-ray diffraction (XRD)* using a Shimadzu LabX XRD-6000 diffractometer operating with Cu Kα radiation (λ = 1.54059 Å) (Shimadzu, Kyoto, Japan). Diffraction patterns were recorded over a 2θ range of 20–80° at a scanning rate of 2°·min^−1^. Phase identification and data analysis were carried out using X’Pert HighScore Plus software, version 2.2c (2.2.3). The average crystallite size was calculated from peak broadening using Scherrer’s equation while structural refinement and quantitative phase analysis were conducted by Rietveld refinement employing the FullProf software package (version 5.10).

Chemical bonding and functional group analysis were investigated by *Fourier transform infrared spectroscopy (FT-IR)* using a Jasco 660 Plus spectrophotometer (JASCO, Tokyo, Japan). Samples were prepared according to the KBr pellet method, and spectra were collected in the 400–4000 cm^−1^ range. Each spectrum represented the average of 16 scans recorded at a spectral resolution of 4 cm^−1^.

Elemental composition was determined by *inductively coupled plasma optical emission spectrometry (ICP-OES)*. For this purpose, 20 μL of MNP suspension were digested with 300 μL of concentrated hydrochloric acid (37%) and maintained at 80 °C for 1 h to ensure complete dissolution. The digested samples were subsequently diluted with Milli-Q water to a final volume of 10 mL and analyzed using an iCAP PRO XP Duo ICP-OES spectrometer (Thermo Scientific, Waltham, MA, USA). Measurements were performed in axial mode for Mn and radial mode for Fe, using the following emission wavelengths: Fe 240.488 nm and Mn 257.610 nm, respectively. All analyses were carried out in triplicate at the Servicio de Análisis Químico (SAI, Universidad de Zaragoza, Zaragoza, Spain).

The *hydrodynamic diameter*, *polydispersity index (PDI)*, and *ζ-potential* were determined after appropriate dilution with ultrapure water using an AMERIGO Particle Size and Zeta Potential Analyzer coupled with a Vasco-Kin contactless remote optical unit (Cordouan Technologies, Pessac, France). ζ-potential measurements were conducted using a carbon electrode, and electrophoretic mobility data were analyzed according to Smoluchowski equation. All measurements were performed at room temperature.

The *electrical conductivity* of ME systems was measured at room temperature using a ProLab 2500 pH/conductivity meter (SI Analytics, Weilheim, Germany) equipped with an LF4134T IDS conductivity probe to evaluate the characteristics of the continuous phase and to confirm the oil-in-water nature of the formulations. Measurements were performed in triplicate, and the results are reported as mean ± standard deviation (SD).

*Magnetic properties* were evaluated at room temperature (22 °C) using a vibrating sample magnetometer (VSM, Lake Shore 7410, San Antonio, TX, USA).

*Particle morphology and size distribution* were examined by transmission electron microscopy (TEM) using a Tecnai T20 transmission electron microscope operating at 200 kV (FEI, Amsterdam, The Netherlands). TEM samples were prepared by depositing 5 µL of diluted MNP suspensions (1 µg Fe·mL^−1^) on a copper grid (200 mesh, Electron Microscopy Sciences, Aname, Madrid, Spain) followed by drying at room temperature before analysis. MNP size distributions were obtained by measuring more than 100 MNPs using Fiji software, version 1.54p. Size distribution statistics were represented in a frequency histogram with a Gaussian fitting.

*SAR measurements* were carried out using a D5 Series AMF applicator (nanoScaleBiomagnetics, Zaragoza, Spain)—with a coil (CAL2) connected to a vacuum pump to achieve thermal isolation of the sample—and a capacitor (120 nF) to evaluate different frequency ranges. MNP samples were prepared at a concentration of 1 mg Fe·mL^−1^ in water. For each measurement, the temperature variation was recorded for 3 min and the SAR was calculated by linear curve fitting within the 30 s right after heating was started. The specific heat absorption rate (SAR) and intrinsic loss power (ILP) were calculated using the following formulas:

$$SAR=c_{mf}\frac{m_{mf}}{m_{Fe}}\left.\frac{∆T}{∆t}\right|\;t\to 0$$

where *C_mf_* is the specific heat capacity of the magnetic fluid, *m_mf_* is the mass of the solution, *m_Fe_* is the mass of iron in the MNPs, and $\frac{∆T}{∆t}$ is the initial linear slope (*t* = 30 s).

$$ILP=\frac{SAR}{f\cdot H^{2}}$$

where *H* is the magnetic field amplitude and *f* is the frequency.

### 2.5. Relaxometric Properties

*Magnetic resonance imaging (MRI)* investigations were conducted to evaluate the potential of the here-reported MNPs as contrast agents. The measurements were carried out using a 1 T small-animal PET–MRI system (nanoScan PET-MRI, Mediso Ltd, Budapest, Hungary), with the static magnetic field (B_0_) calibrated to the resonance frequency of water protons.

Nanoparticle dispersions were initially prepared in phosphate-buffered saline (PBS, pH 7.4) and subjected to ultrasonication for 15 min to ensure homogeneous distribution. Defined volumes of these stock suspensions were subsequently mixed with a 1% (*w*/*v*) agarose solution prepared in PBS (pH 7.4), yielding final Fe concentrations in the range of 0.006–0.1 mM. Considering that the samples consist of Mn-doped magnetite with varying doping levels (1–10%), all dilutions were adjusted to maintain equivalent Fe content across the series. For imaging, 3 mL of each prepared sample were transferred into individual wells of an 8-well plate, while an additional well containing only 1% agarose gel served as a reference. The same preparation and measurement protocol were applied for the ME-based systems, ensuring direct comparability of the relaxometric data.

MRI signal contrast is governed by the longitudinal (*T*_1_) and transverse (*T*_2_) relaxation processes, which were quantified using standard acquisition sequences. Longitudinal relaxation times (*T*_1_) were determined using two-dimensional gradient echo (T1-GRE) sequences with the following parameters: repetition time (*TR*) = 381.9 ms, echo time (*TE*) = 3.8 ms, number of excitations (NEX) = 2, slice thickness = 3 mm, slice gap = 1 mm, frequency resolution = 0.5 mm, phase resolution = 0.735 mm, and variable flip angles of 10°, 20°, 60°, and 70°. Circular regions of interest (ROI, 10 mm diameter) were defined within each sample, and the corresponding mean signal intensities were extracted. The *T*_1_ values were estimated using a two-point method (flip angles of 10° and 60°), according to the following:

$$\ln\left[\frac{\left(I_{1}sin\theta_{2}-I_{2}sin\theta_{1}\right)}{{(I}_{1}sin\theta_{2}cos\theta_{1}-I_{2}sin\theta_{1}cos\theta_{2})}\right]=\frac{-TR}{T_{1}}$$

where *I*_1_ and *I*_2_ denote the measured signal intensities, *TR* is the repetition time, and *θ*_1_ and *θ*_2_ represent the applied flip angles expressed in radians.

The *T*_2_ relaxation times were obtained from two-dimensional spin echo (T2-SE) sequences using *TR* = 1896 ms and echo times (*TE*) of 20, 40, 80, and 120 ms. The decay of the signal intensity was fitted using the exponential relation:

*I = A∙e^(−TE/T2)^*,

where *I* represents the mean signal intensity in ROI, *A* is the initial signal intensity, and *TE* is the echo time.

The longitudinal (*r*_1_) and transverse (*r*_2_) relaxivities were determined from the slopes of the linear dependence of *R*_1_ (1/*T*_1_) and *R*_2_ (1/*T*_2_) on the iron concentration.

### 2.6. Drug Release Studies

Release studies of DOX were conducted using the dialysis technique with phosphate buffer (PB, pH 7.4) as the release medium. The experiments were performed at 37 °C under continuous magnetic agitation (100 rpm) using two Heidolph MR Plug magnetic stirrers (Heidolph Instruments GmbH & Co. KG, Schwabach, Germany). Aliquots of the formulations (3 mL of suspension) were placed into dialysis membranes with a molecular weight cut-off of 14.000 Da (Sigma-Aldrich, St. Louis, MO, USA) and subsequently immersed in 100 mL of PB. At predefined time intervals, 2 mL samples were withdrawn from the external release medium and immediately replaced with an equivalent volume of fresh PB to maintain constant volume and sink conditions. All experiments were performed in duplicate (Figure S1).

The amount of DOX released into the PB release medium was quantified by UV–Vis spectrophotometry using a Shimadzu UV–Vis Ultimate-1900i spectrophotometer (Shimadzu Corporation, Kyoto, Japan). Absorbance measurements were recorded at 480 nm, corresponding to the characteristic absorption maximum of DOX in aqueous media. Quantitative analysis was performed using a calibration curve prepared in the same buffer (Figure S2).

## 3. Results and Discussions

### 3.1. Design and Selection of Mn-Doped Magnetic Nanoparticles

In the present work, Mn was introduced at nominal levels of 1%, 5%, and 10% relative to the total Fe content while deliberately preserving the Fe^2+^/Fe^3+^ molar ratio characteristic of magnetite (Fe_3_O_4_) throughout the synthesis. This approach differs from classical stoichiometric replacement strategies and enables the evaluation of how progressive Mn incorporation influences structural and functional properties. The systematic screening of these compositions allows for identification of a formulation that provides a balanced compromise between magnetic heating potential and compatibility with subsequent drug loading and stable incorporation into oil-in-water ME matrices.

**Figure 1:**
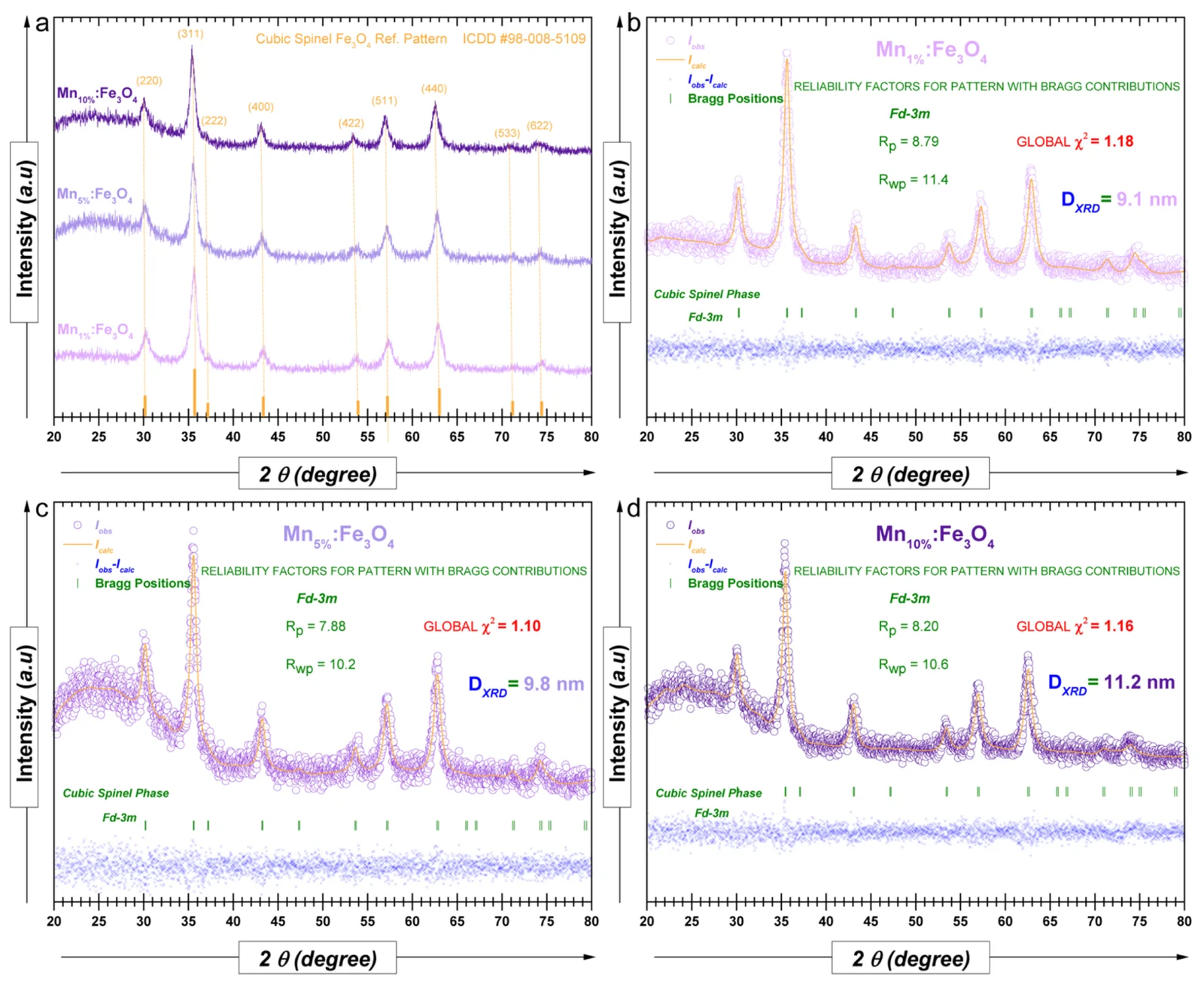
(**a**) Superimposed powder XRD patterns of all doped samples (Mn_1%_, Mn_5%_, Mn_10%_). Le Bail refinement was performed using the FullProf software on the powder XRD data of (**b**) Mn_1%_:Fe_3_O_4_; (**c**) Mn_5%_:Fe_3_O_4_; and (**d**) Mn_10%_:Fe_3_O_4_. Profile fitting was performed through the iterative refinement process of the experimental XRD patterns with the calculated diffraction patterns generated from the proposed crystal structure models (orange line). Bragg positions are presented in green bars, and the difference of the experimental XRD patterns with the calculated diffraction patterns is represented in blue color.

The crystalline structure and phase purity of Mn-doped MNPs (Mn_1%_, Mn_5%_, and Mn_10%_) were investigated by XRD analysis. As shown below, the superimposed diffraction patterns (Figure 1a) display the characteristic reflections of the cubic spinel structure of magnetite (Fe_3_O_4_), indexed to (220), (311), (400), (422), (511), and (440) crystallographic planes. No additional peaks that might correspond to manganese oxide phases or other crystalline iron oxide polymorphs are detected. The absence of secondary reflections indicates that Mn incorporation does not induce phase segregation, even at 10 mol% doping. All samples can be indexed to a single cubic phase belonging to the Fd–3m space group, confirming the preservation of the inverse spinel phase. Moreover, Le Bail refinements performed using the FullProf software further confirm the structural integrity of the doped systems.

The refinement profile (Figure 1b–d) shows excellent agreement between experimental and calculated patterns, with low residuals and minimal differences. In fact, the reliability factors (R_p_ = 7.88 ÷ 8.79%, R_wp_ = 10.2–11.4%, χ^2^ = 1.10 ÷ 1.18) demonstrate the robustness of the structural model and the high crystallinity of the samples. Given these points, it is clear that Mn doping up to 10% does not disrupt long-range crystallographic order, and all samples are identified as the cubic spinel phase. A systematic and monotonic increase in lattice parameter is observed with increasing Mn content (Table 1). The refined lattice parameter increases from 8.3566(7) Å (Mn_1%_) to 8.3721(5) Å (Mn_5%_), and further to 8.3984(7) Å (Mn_10%_), accompanied by a corresponding expansion of the unit cell volume from 583.5(8) Å^3^ to 592.3(8) Å^3^. As a result, the progressive lattice expansion strongly supports successful Mn incorporation into the spinel lattice. Considering ionic radii, Mn^2+^ in octahedral coordination (~0.83 Å) is larger than Fe^2+^ (~0.77 Å) and Fe^3+^ (~0.645 Å). Therefore, the substitution of Fe^2+^ by Mn^2+^ at octahedral sites is expected to generate isotropic lattice expansion, as experimentally observed (Table 1). The observed structural behavior becomes particularly meaningful when compared to our previously reported low-level Co-doped Fe_3_O_4_ system. As highlighted in that work [31], the lattice parameter evolution with increasing Co content was non-linear and did not follow a simple type trend. On balance, those deviations were interpreted as evidence of partial non-substitutional incorporation and local lattice distortions. By contrast, the Mn-doped samples presented here exhibit a clear and progressive lattice expansion consistent with a substitutional mechanism. In short, the crystallographic response to Mn incorporation appears more regular and predictable.

**Figure 2:**
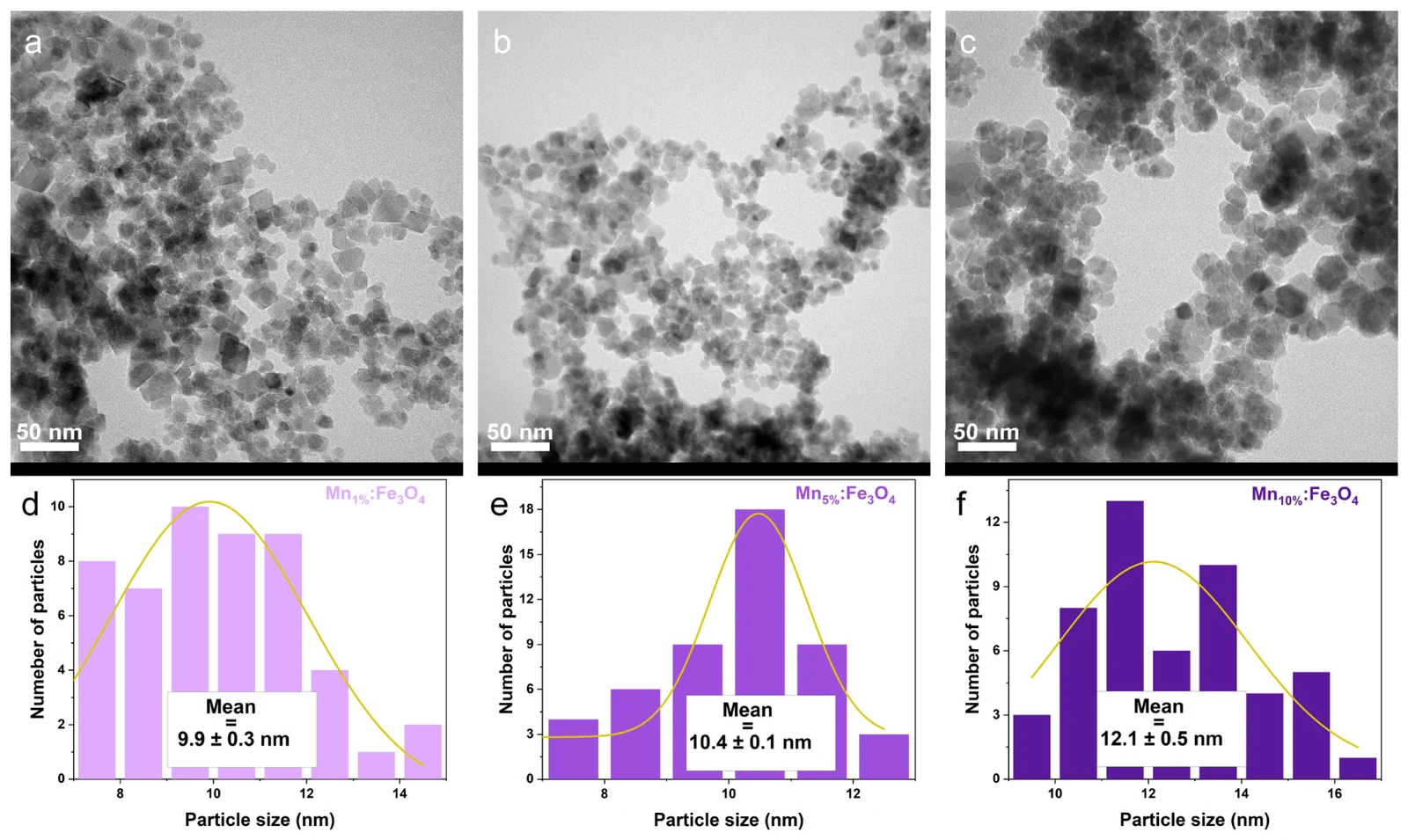
TEM images (scale bar 50 nm) and particle size distribution of (**a**) Mn_1%_:Fe_3_O_4_ with (**d**) mean size of 9.9 nm, (**b**) Mn_5%_:Fe_3_O_4_ with (**e**) mean size of 10.4 nm, (**c**) Mn_10%_:Fe_3_O_4_ with (**f**) mean size of 12.1 nm.

The TEM images (Figure 2a–c) and corresponding histograms (Figure 2d–f) further substantiate this trend, illustrating the morphology and size distribution of the Mn-doped MNPs.

The particles exhibit a polyhedral morphology with good size dispersion, consistent with the structural homogeneity revealed by the diffraction analysis. A slight increase in mean particle size from 9.9 to 12.1 nm is observed with increasing Mn content, suggesting that Mn incorporation subtly affects the nucleation and growth kinetics during synthesis. This difference can be rationalized by considering the distinct electronic configurations and crystal field stabilization energies of Mn^2+^ (high-spin *d*^5^) and Co^2+^ (*d*^7^). In essence, the half-filled *d*-shell of Mn^2+^ is electronically symmetric and less prone to inducing Jahn–Teller-type distortions, which may explain the more uniform structural evolution observed here. Consequently, the Mn-doped system maintains isotropic lattice expansion without evidence of structural perturbation. In light of these facts, the contrast between Mn and Co doping highlights to some extent the dopant-dependent nature of cation incorporation in magnetite (Fe_3_O_4_). Also, the crystallite size shows a gradual increase with Mn doping, evolving from 9.1 nm (Mn_1%_) to 9.8 nm (Mn_5%_) and 11.2 nm (Mn_10%_). This moderate increase sustains that Mn incorporation slightly influences nucleation and growth kinetics during precipitation, possibly by modifying surface energy or reducing defect density within the spinel domains. Nevertheless, the growth process remains controlled and does not indicate secondary phase formation. Taken together, and particularly in comparison with our previous study focused on a Co-doped system mentioned above, the Mn-doped nanoparticles investigated in the present study display a more uniform and homogeneous structural response. Ultimately, this distinction is significant because it suggests that Mn doping provides a structurally stable platform for tuning magnetic and relaxometric properties without introducing local distortions observed for the Co system. The crystallographic results observed here establish a solid foundation for correlating structural modifications with the functional behavior discussed in the following sections.

**Table 1:** Refined structural parameters and reliability factors for Mn low-level doped Fe_3_O_4_ pure cubic spinel phases.

Sample	Sample Composition by PXRD (Crystalline Phase Identified/%)		Space Group	Direct Cell Parameters (Å)	Direct Cell Volume (Å^3^)	Reliability Factors			Size (nm)
						R_p_	R_wp_	χ2	
**Mn_1%_**	Spinel Phase	100	Fd–3m	8.3566 (7)	583.5 (8)	8.79	11.4	1.18	9.1
**Mn_5%_**	Spinel Phase	100	Fd–3m	8.3721 (5)	586.8 (3)	7.88	10.2	1.10	9.8
**Mn_10%_**	Spinel Phase	100	Fd–3m	8.3984 (7)	592.3 (8)	8.20	10.6	1.16	11.2

**Figure 3:**
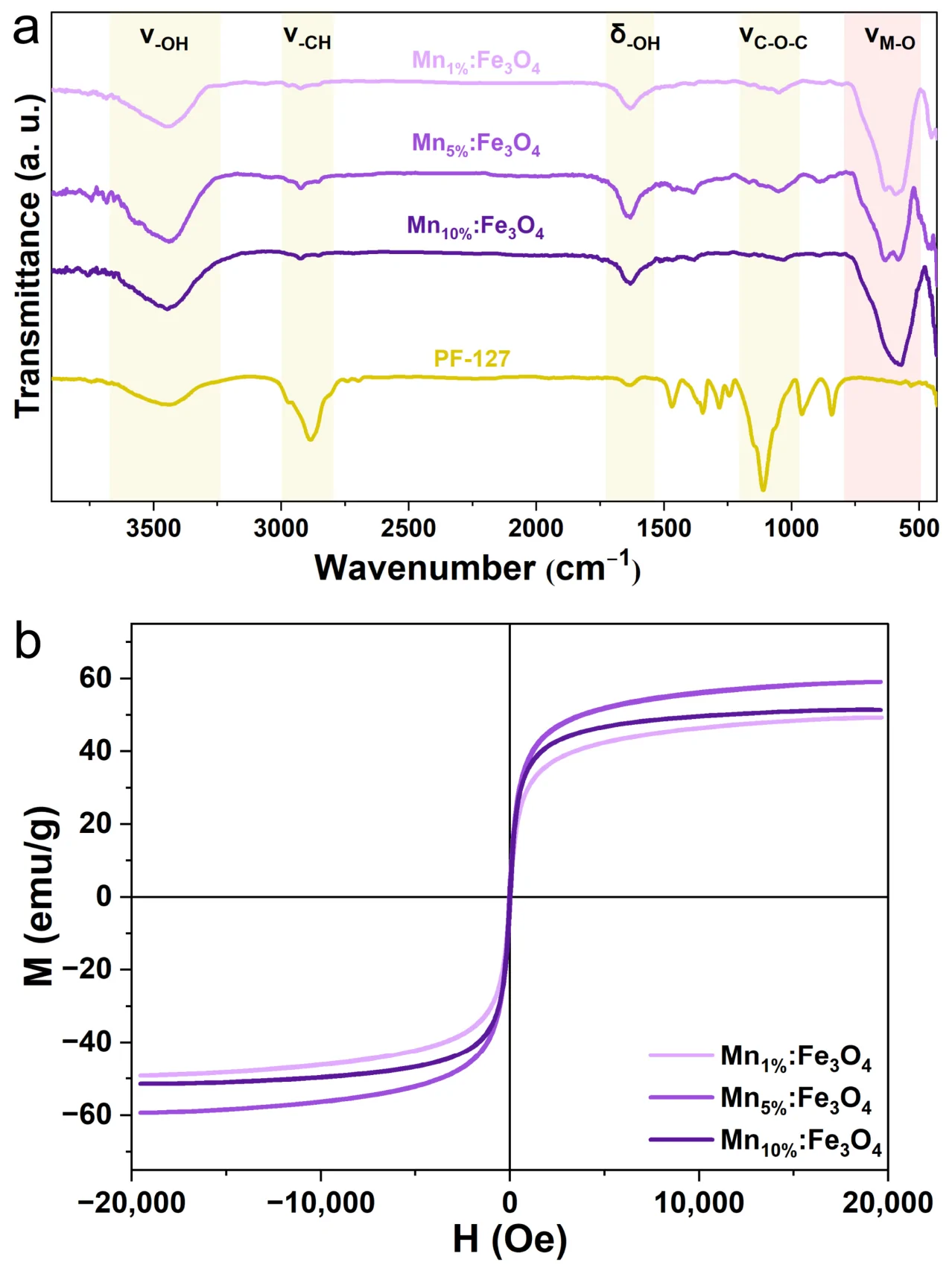
(**a**) FT-IR spectra of PF-127 and Mn-doped nanoparticles Mn_1%_:Fe_3_O_4_, Mn_5%_:Fe_3_O_4_, and Mn_10%_:Fe_3_O_4_. (**b**) Magnetic hysteresis loops (M–H curves) of the Mn-doped ferrite samples measured at room temperature.

FT-IR spectroscopy was employed to examine the vibrational signatures associated with the metal–oxygen lattice and to assess the surface interactions between the nanoparticles and the Pluronic F-127 stabilizing polymer. All samples (Figure 3a) exhibit a broad absorption band in the 3400–3500 cm^−1^ region, assigned to ν-OH stretching vibrations associated with surface hydroxyl groups and adsorbed water, a typical feature of Fe_3_O_4_ nanoparticles synthesized in aqueous media [32]. The bands observed in the 2850–2950 cm^−1^ and 1100–1150 cm^−1^ regions correspond to ν-CH and C–O–C vibrations, respectively, confirming the adsorption of Pluronic F-127 on nanoparticles’ surfaces and supporting effective steric stabilization [33,34].

All Mn-doped samples exhibit an absorption band in the 1600–1650 cm^−1^ region, attributed to the bending vibration of molecular water (δ-OH) adsorbed on the surface of nanoparticles, a common feature of hydrated iron oxide-based nanomaterials. In the low-wavenumber region (600–450 cm^−1^), all compositions display the characteristic broad metal–oxygen stretching vibrations [35,36]. Importantly, this region reflects the collective contribution of (Fe/Mn)-O lattice vibrations rather than distinct Fe–O and Mn–O modes, indicating that Mn incorporation does not generate separate oxide domains detectable by FT-IR. The persistence of this band across the doping series confirms that the spinel iron oxide framework is preserved. Minor variations of the band shape with increasing Mn content suggest local modifications of the Me–O bond environment consistent with dopant incorporation during crystal growth rather than structural disruption or secondary phase formation.

Although the FT-IR spectra confirm the preservation of a spinel lattice and the absence of detectable secondary oxide phases, vibrational spectroscopy does not provide quantitative information regarding the amount of Mn incorporation within the nanoparticles. Elemental analysis by ICP-OES was therefore performed to determine the actual dopant concentration achieved during synthesis. The obtained results confirm the effective and controlled incorporation of Mn into the MNPs across the investigated series (Table 2). The experimentally determined Mn concentrations (0.069, 0.304, and 0.586 mg·mL^−1^ for Mn_1%_, Mn_5%_, and Mn_10%_, respectively) follow the nominal trend (0.074, 0.343, and 0.685 mg·mL^−1^), indicating reproducible modulation of dopant levels. A gradual decrease in incorporation efficiency is observed with increasing Mn content despite the preservation of the Fe^2+^/Fe^3+^ ratio during synthesis. This behavior reflects the competitive incorporation of Mn^2+^ into the spinel lattice during coprecipitation. Because the iron redox ratio was deliberately preserved, Mn introduction does not occur via the displacement of Fe from the Fe_3_O_4_ lattice; instead, Mn^2+^ competes with iron cations during nucleation and crystal growth, leading to the formation of a mixed ferrite composition. Iron is not incorporated into the nanoparticle lattice but rather remains in the solution and is subsequently removed during the washing process. This behavior accounts for the experimentally observed proportional reduction in Fe content as the Mn concentration increases. The correlated decrease in Fe and increase in Mn, together with the absence of secondary phases in FT-IR analysis, supports dopant integration within a preserved spinel framework rather than the formation of segregated MnO_x_ domains.

Dynamic light scattering (DLS) measurements were also carried out to examine how Mn incorporation affects the hydrodynamic size and colloidal dispersion behavior of the nanoparticle suspensions. The results of these measurements revealed hydrodynamic diameters of 212 nm, 214 nm, and 316 nm for Mn_1%_, Mn_1%_, andMn_10%_, respectively. The very similar sizes obtained for Mn_1%_ and Mn_5%_ suggest that low and moderate Mn incorporation does not significantly affect the dispersion behavior of the nanoparticles. The Mn_10%_ sample shows a slight increase in nanoparticle size, reaching 316 nm. Since DLS measures the hydrodynamic diameter, which includes the particle core together with the surrounding solvent layer and any adsorbed stabilizing polymer, the larger hydrodynamic diameter likely reflects partial nanoparticle aggregation or changes in interparticle interactions rather than the growth of the primary nanocrystals [37,38].

Additional insight into colloidal behavior is provided by ζ-potential measurements. The values increased from +2.56 mV for Mn_1%_ to +7.68 mV for Mn_5%_ and +20.36 mV for Mn_10%_, indicating the shift of the surface charge toward more positive values with the increase in Mn content (Table 2). In ferrite systems, the substitution of Fe by divalent metal ions, such as Mn^2+^, can influence the chemical environment of surface hydroxyl groups, which play a key role in determining surface charge and electrokinetic behavior. As introduced above, the characteristic Fe^2+^/Fe^3+^ ratio for magnetite was deliberately maintained at 1/2 for the samples discussed herein, while Mn was introduced in increasing nominal amounts during coprecipitation. As confirmed by XRD analysis, all samples retained the inverse spinel structure with a progressive lattice expansion, indicating the incorporation of Mn^2+^ within the ferrite lattice. Under these conditions, Mn does not replace Fe in a strict stoichiometric substitution but instead competes with iron cations during nucleation and growth, leading to a mixed ferrite composition in which Mn^2+^ is incorporated into the spinel framework. This compositional modification can alter the distribution and protonation behavior of surface hydroxyl groups, thereby affecting the electrokinetic potential of the nanoparticles [39]. Despite the observed increase in ζ-potential, the measured values remain below ±30 mV, a threshold typically associated with the strong electrostatic stabilization of colloidal dispersions, indicating that electrostatic repulsion alone provides only moderate colloidal stability [40]. The moderate ζ-potential values are consistent with DLS results, showing hydrodynamic clustering at higher Mn content. This behavior may be attributed to molecules adsorbed on the particle surface during synthesis.

The magnetic behavior of Mn-doped Fe_3_O_4_ nanoparticles was evaluated by vibrating sample magnetometry (VSM) at room temperature. The corresponding hysteresis loops, together with the extracted magnetic parameters (M_s_, M_r_, H_c_, M_r_/M_s_), are presented in Figure 3b and Table 3.

All samples show a narrow hysteresis, typical of soft magnetic materials, with very low values of remanence and coercivity, confirming a predominantly superparamagnetic-like behavior at room temperature. The undoped magnetite shows a saturation magnetization of 58.6 emu·g^−1^, coercivity of 17.2 Oe, and M_r_/M_s_ ratio of 0.039, in agreement with the values found in the literature for nanoparticles with a well-crystallized spinel structure [39,41]. The introduction of Mn^2+^ ions into the spinel structure leads to a nonlinear evolution of M_s_. An initial decrease is observed at Mn_1%_ (49.23 emu·g^−1^), followed by an increase above the Fe_3_O_4_ value at Mn_5%_ (59.21 emu·g^−1^), and at 10% Mn, the magnetization decreases moderately to 55.1 emu·g^−1^. This trend correlates directly with XRD observations, which show a systematic expansion of the lattice parameter, confirming the preferential substitution of Fe^2+^ ions from octahedral sites by Mn^2+^ with a larger ionic radius. For low and moderate doping levels (1–5%), the substitution maintains the cationic spinel architecture, and the high magnetic moment of Mn^2+^ (*d*^5^ configuration, high spin) can locally strengthen the super-exchange interactions between A and B sublattices through changes in cation distribution and local magnetic moments, which justifies the increase in magnetization at 5% Mn. At 10% Mn, however, the increase in interionic distances and the dilution of the Fe^2+^–O–Fe^3+^ paths on an octahedral sublattice lead to a partial decrease in magnetic order, resulting in a slight reduction in M_s_. The coercivity (H_c_) shows a moderate evolution, generally increasing and ranging from 12.41 Oe (Mn_1%_) to 17.37 Oe (Mn_5%_) and 23.25 Oe (Mn_10%_). The decrease in the value at Mn_1%_ may indicate a reduction in the effective magnetocrystalline anisotropy, possibly related to particle size and cation distribution, corresponding to small particles (9.1 nm). At higher doping, the increase in coercivity reflects both the lattice expansion, which affects the exchange energy, and the higher crystal size, but the values remain within the limits of the superparamagnetic range. The very low M_r_/M_s_ ratios (0.022–0.044) indicate weak magnetic blocking and a predominantly superparamagnetic-like behavior for all samples. Comparison of these results with our previously reported Co-doped system highlights the fundamental differences between the two dopants. While Co^2+^ (*d*^7^) induced a pronounced increase in magnetocrystalline anisotropy, generating visible hysteresis and large increases in coercivity even at low doping, Mn^2+^ (*d*^5^, symmetric) produces much more uniform changes. This fact explains the linear structural evolution of the lattice parameter without local distortions and the maintenance of a very narrow hysteresis for all Mn-doped samples. Overall, Mn doping proves to be a structurally benign and magnetically predictable method of modifying the properties of Fe_3_O_4_. It allows for the adjustment of saturation magnetization without compromising superparamagnetic behavior, which is essential for biomedical applications such as MRI or magnetic hyperthermia.

**Figure 4:**
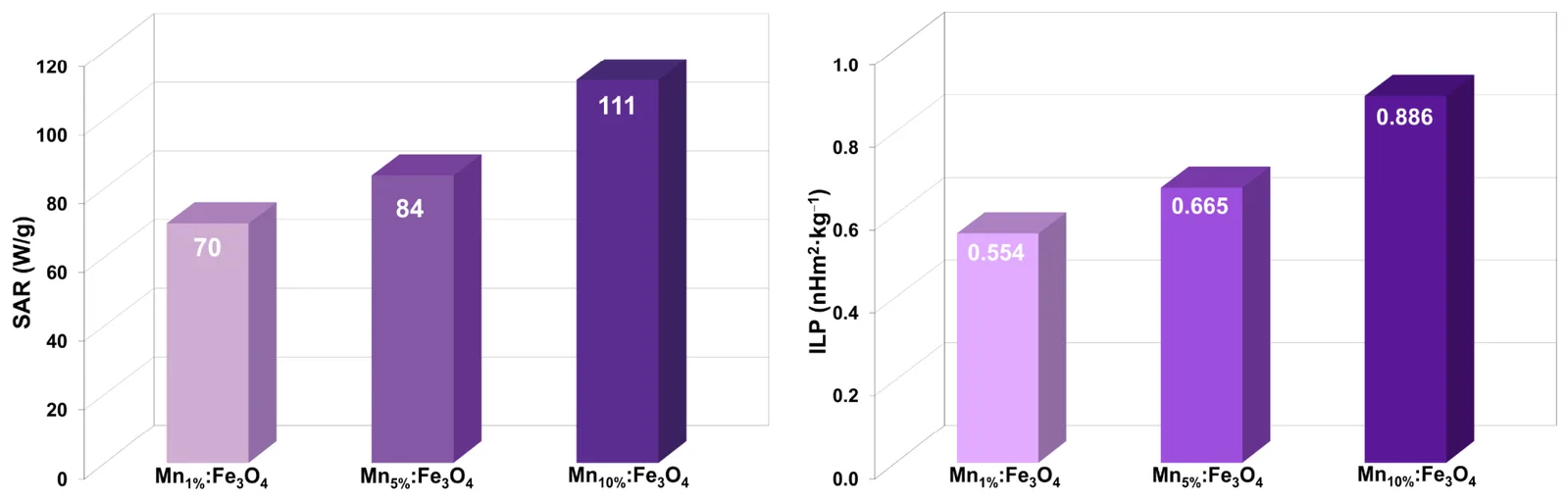
Specific absorption rate (SAR) and intrinsic loss power (ILP) values of Mn-doped ferrite nanoparticles, measured at 497 kHz and 200 G, show an increase in heating efficiency with increasing Mn content.

The SAR was determined to quantify the heating efficiency of the Mn-doped nanoparticles and to evaluate whether Mn incorporation enhances HT performance. SAR was therefore evaluated under fixed AMF conditions of 497 kHz and 200 G (≈16 kA·m^−1^) at a concentration of 1 mg Fe·mL^−1^, using the initial 30 s time interval of heating to ensure measurements within the linear temperature-rise regime (Figure 4). Under these conditions, SAR increases progressively across the series, reaching 111.47 W·g^−1^ for Mn_10%_ compared to 83.60 W·g^−1^ for Mn_5%_ and 69.67 W·g^−1^ for Mn_1%_. This SAR increase does not follow the non-linear change observed for M_s_. Instead, it correlates with coercivity and the M_r_/M_s_ ratio, indicating that heating efficiency under these conditions is influenced more by magnetic anisotropy and particle–particle interactions rather than saturation magnetization alone. For Mn-doped ferrites specifically, dopant-induced cation redistribution within the spinel lattice can modify super-exchange interactions and alter the effective magnetic anisotropy. Yan et al. [42] demonstrated that Mn substitution induces structural disorder and modifies magnetic parameters, including M_s_ and coercivity. Similar observations have been reported for compositionally tuned ferrites where moderate Mn incorporation enhances heating performance despite a non-linear evolution of M_s_ [27,43].

These values fall within the typical range reported for iron-oxide-based nanoparticles used in magnetic HT. Previous studies indicate that Fe_3_O_4_ nanoparticles exposed to alternating magnetic fields of 100–500 kHz and 10–40 kA·m^−1^ commonly exhibit SAR values of approximately 100–300 W·g^−1^, depending on particle size distribution, magnetic anisotropy, and interparticle interactions [44,45,46]. It should also be noted that a direct comparison of SAR values between studies remains challenging due to differences in experimental parameters, such as magnetic field calibration, nanoparticle concentration, and thermal losses [12,47].

Beyond magnetic heating, Mn incorporation is also relevant from a relaxation standpoint in the context of MRI. Since dopant-induced modifications of local magnetic fields and anisotropy can influence proton relaxation behavior, the assessment of MRI-related properties is required to determine whether the composition selected for HT also offers imaging functionality. The relaxometric characteristics of Mn-doped nanoparticles are therefore examined within the framework of multifunctional formulation design, with the results discussed alongside those obtained for the magnetic ME system (vide infra), enabling direct evaluation of the confinement and matrix effects on relaxometric performance.

The comparative evaluation of Mn-doped series demonstrates that compositional variation systematically influences magnetic, calorimetric, and relaxometric properties. Although Mn_5%_ exhibits the highest saturation magnetization and Mn_1%_ exhibits the largest transverse relaxivity, the overall functional performance cannot be determined from a single parameter. Instead, the data indicate that Mn_10%_ achieves the most favorable balance between magnetic anisotropy, heating efficiency, and preserved *T*_2_ contrast capability, making this composition the optimal one for incorporation into the ME matrix. Moreover, Mn_10%_ demonstrated the highest SAR and ILP, which was considered to be particularly important because magnetic HT, together with DOX delivery, represents the therapeutic component of the proposed nanoformulation.

The next section addresses the integration of Mn_10%_ nanoparticles into the optimized oil-in-water ME system and the preparation of the corresponding DOX@MNP formulation.

### 3.2. Microemulsion Integration of Mn_10%_:Fe_3_O_4_ and DOX@Mn_10%_:Fe_3_O_4_ Controlled Release and Therapeutic Implications

Following the identification of Mn_10%_:Fe_3_O_4_ as the optimal composition, DOX loading was evaluated through passive adsorption. The analysis of the supernatant after magnetic separation showed values of EE% and DLC% of 47.2% and 7.7%, respectively, confirming the successful adsorption of DOX onto the MNPs surface under mild conditions. The resulting DOX-loaded MNPs (DOX@MNPs) were subsequently used as the magnetic-drug component for ME preparation. These nanoparticles were incorporated into the previously optimized oil-in-water ME formulation (F9), which served as the base composition for all formulations investigated in this study.

To evaluate the individual and combined contributions of the carrier, magnetic phase, and the drug, four formulations were prepared: (i) blank ME, (ii) DOX-loaded ME without MNPs (DOX@ME), (iii) MNP-loaded ME without the drug (MNP-ME), and (iv) a formulation containing DOX-adsorbed MNPs (DOX@Mn_10%_-ME). This design enables direct comparison of the roles of each component and their combined behavior within a multifunctional nanoplatform.

Blank MEs were prepared by mixing the oil phase, surfactant/co-surfactant mixture (S_mix_), and aqueous phase according to the predefined F9 mass ratios, under continuous magnetic stirring at room temperature until a transparent and optically isotropic dispersion was obtained, indicating ME formation. For DOX@ME, solid DOX hydrochloride was first dispersed in the oil phase to ensure homogeneous distribution. The aqueous phase was then added dropwise under stirring, allowing for the spontaneous formation of ME.

For formulations containing MNPs, the as-synthesized aqueous suspension of Mn_10%_ was used as the aqueous phase without drying or redispersion in order to preserve its colloidal properties. The DOX@Mn_10%_-ME formulation was prepared in the same manner using the suspension of DOX-loaded nanoparticles. All formulations showed no signs of turbidity, phase separation, or sedimentation. The F9 composition was kept constant to ensure that any observed differences arise from the presence of nanoparticles and/or the drug rather than from changes in formulation ratios. Samples were allowed to equilibrate at ambient conditions before further physicochemical characterization.

To determine whether the incorporation of DOX, Mn_10%_ MNPs, or DOX-loaded nanoparticles influences the internal organization of MEs, the colloidal structure of the formulations was investigated using DLS, electrical conductivity, and ζ-potential measurements (Table 4). DLS provides information on the hydrodynamic diameter and size distribution of dispersed structures, whereas conductivity measurements allow for the identification of the continuous phase and verification of the oil-in-water character of the formulations [48,49]. The combined analysis of these parameters enables the assessment of potential changes in droplet organization, aggregation behavior, or structural transitions induced by drug or nanoparticle incorporation.

The physicochemical characterization indicates that the incorporation of DOX and Mn_10%_ MNPs preserve the oil-in-water organization of F9 ME while inducing small changes in droplet structure. The blank formulation exhibited a conductivity of 20.76 μS·cm^−1^, which remained almost unchanged after nanoparticle incorporation (19.70 μS·cm^−1^), but slightly increased in the DOX-containing formulation (21.50 μS·cm^−1^). The similarity of these values across all formulations confirms the persistence of a continuous aqueous phase. Such stability in conductivity following nanoparticle incorporation is consistent with previous observations for oil-in-water MEs, where structural integrity is reflected by relatively constant conductivity values as long as the aqueous phase remains continuous [48,50,51].

**Table 4:** Electrical conductivity, hydrodynamic diameter (D_h_, *n* = 3), polydispersity index (PDI, *n* = 3), and ζ-potential values (*n* = 6).

Sample	Conductivity (μS/cm)	DLS		
		D_h_ (nm)	PDI	ζ-Potential (mV)
*Freshly prepared*				
**ME**	20.76 ± 0.416	170 ± 1.733	0.292 ± 0.031	−21.0 ± 0.599
**DOX@Mn_10%_-ME**	19.70 ± 0.200	270 ± 3.092	0.314 ± 0.023	−15.0 ± 1.280
**DOX@ME**	21.50 ± 0.100	126 ± 1.469	0.277 ± 0.018	−17.4 ± 1.097
*After 6 months*				
**ME**		188 ± 5.340	0.3642 ± 0.008	−23.0 ± 0.660
**DOX@Mn_10%_-ME**		281 ± 4.836	0.2989 ± 0.024	−17.5 ± 1.250
**DOX@ME**		249 ± 3.534	0.3844 ± 0.007	−24.0 ± 0.605

Note: Replicates represent repeated measurements of the same sample.

DLS measurements further support the preservation of the ME’s structure after DOX and MNPs incorporation. The blank ME exhibited a mean hydrodynamic diameter of 170 nm with a PDI of 0.292, consistent with a relatively homogeneous dispersed phase. In the absence of nanoparticles, the incorporation of DOX led to a reduction in droplet size to 126 nm (PDI 0.277), suggesting that the drug influences interfacial organization. This decrease is consistent with the ability of amphiphilic molecules such as DOX to partition at the oil-water interface, modify interfacial packing, and alter droplet curvature, thereby reducing the apparent hydrodynamic diameter. Similar behavior has been reported in drug-loaded ME systems in which solubilized compounds affect interfacial tension and packing constraints [52,53,54,55].

The DOX@Mn_10%_-containing ME displayed a larger mean hydrodynamic diameter of 270 nm with a PDI of 0.314. Because MNPs were introduced in an aqueous suspension rather than incorporated within the oil droplets as a solid, this increase is unlikely to originate from direct droplet loading. Instead, it is more plausibly attributed to changes in the physico-chemical environment of the continuous phase, induced by the presence of dispersed nanoparticles and their associated polymer corona. In systems containing hydrophilic MNPs, the particles typically remain dispersed within the aqueous phase rather than adsorbing at the droplet interface. The magnetic clusters formed under external fields primarily influence the surrounding fluid motion rather than generating stable droplet-particle agglomerates [56]. Consequently, the moderate increase in apparent particle size most likely reflects changes in the surrounding colloidal environment and an increase in the effective hydrodynamic radius instead of true enlargement of the oil droplets. The absence of turbidity or phase separation further indicates that the system remains colloidally stable, suggesting that size increase arises from structural adjustment within the dispersion and not from ME’s destabilization.

To further evaluate the colloidal characteristics of the formulations, the droplets’ surface charge was assessed by ζ-potential measurements. The ζ-potential values remained moderately negative for all formulations, with values of −21.0 mV for the blank ME, −17.7 mV for DOX-ME, and −15.0 mV for DOX@Mn_10%_-ME. These results indicate that colloidal stability is maintained after the incorporation of both drug and nanoparticles. Lower negative ζ-potential observed for the MNPs-containing formulation is consistent with the presence of MNPs in the aqueous phase, which exhibit a positive ζ-potential (+20.36 mV) and can partially compensate the negative surface charge of the droplets through electrostatic interactions within the continuous phase.

It is worth underlining that after six months of storage at 4 °C, the DLS measurements showed minor changes in the hydrodynamic diameter and PDI of the unloaded ME and the DOX@Mn_10%_-ME formulation, indicating the preservation of their colloidal characteristics under the investigated storage conditions. On the contrary, an increase in size was noticed for the DOX-loaded ME without MNPs. However, the PDI < 0.4 still shows a relatively narrow size distribution with moderate polydispersity [57]. Also, no visible phase separation or sedimentation was observed for the stored samples, supporting stability over time.

The structural organization of the F9 ME has been previously explained using complementary spectroscopic techniques, particularly DOSY NMR, which demonstrated that the formulation consists of a normal oil-in-water (O/W) ME, with oil droplets dispersed within the continuous aqueous phase and stabilized by a surfactant/co-surfactant interfacial layer [25]. To this ME, a suspension of MNPs was added. It is worth mentioning that the MNP suspension was not put into direct contact with the pre-formed ME. Instead, the MNPs were first dispersed in the aqueous phase used for ME preparation, ensuring the prewetting of the Pluronic F127 dispersant coating the MNPs. Consequently, at the initial stage of the preparation of ME, the MNPs were exclusively localized within the continuous aqueous phase. On the other hand, Pluronic triblock copolymer coatings generally make metal and metal–oxide nanoparticles dispersible in water, but still interfacially active [58]. This is explained by their amphiphilic nature, consisting of hydrophobic poly(propylene oxide) (PPO) and hydrophilic poly(ethylene oxide) (PEO) segments [59]. This architecture provides coated nanoparticles with an affinity for both aqueous and lipidic environments. Specifically, the PPO segments can interact with the lipid-rich domains of the ME, whereas the hydrated PEO chains remain solvated within the continuous aqueous phase. As the ME assembles, the formation of a large oil–water interfacial area becomes favorable for the interfacial adsorption of MNPs, as well. Therefore, a structural arrangement with MNPs dispersed in the water phase as well as adsorbed at the water–oil interface can be envisaged for the MNPs dispersed in ME, which improves their colloidal stability. This achievement is supported by the preservation of the dispersibility of MNPs for a longer period as compared to the simple suspension of F127-coated MNPs [60,61]. Therefore, besides stabilizing the nanoparticles in the aqueous phase, the F127 coating can also promote their adsorption at the oil–water interface, improving the long-term stability. Based on this discussion, it can be affirmed that although the nanoformulation developed herein is not truly a Pickering system due to the presence of the surfactant [62], the existence of F127 on the MNPs’ surface promotes the formation of a system exhibiting a Pickering-like behavior or a system with hybrid interfacial stabilization.

The physicochemical characterization is consistent with this structural model, particularly with the presence of a constant part of MNPs mainly at the interface, as discussed further. The electrical conductivity remains essentially unchanged after nanoparticle incorporation, indicating that the continuous aqueous phase characteristic of the O/W ME is preserved. Simultaneously, DLS measurements reveal only a moderate increase in hydrodynamic diameter, suggesting that the incorporation of the DOX@Mn_10%_ nanoplatform results in a limited expansion or reorganization of the dispersed droplets rather than disruption of the ME architecture. Such behavior supports the localization of MNPs within the interfacial region, too. Collectively, the structural and physicochemical findings reinforce a structural model in which the F9 ME retains its original O/W organization after loading with MNPs, while the PF127-coated MNPs are proposed to be preferentially localized at the droplet interface, forming an integrated hybrid nanostructure. This arrangement preserves the continuous aqueous network required for colloidal stability while simultaneously combining the drug delivery function of the ME with the magnetic responsiveness of the MNPs, thereby underpinning its potential as a multifunctional platform for medical applications [12,25,61].

**Figure 5:**
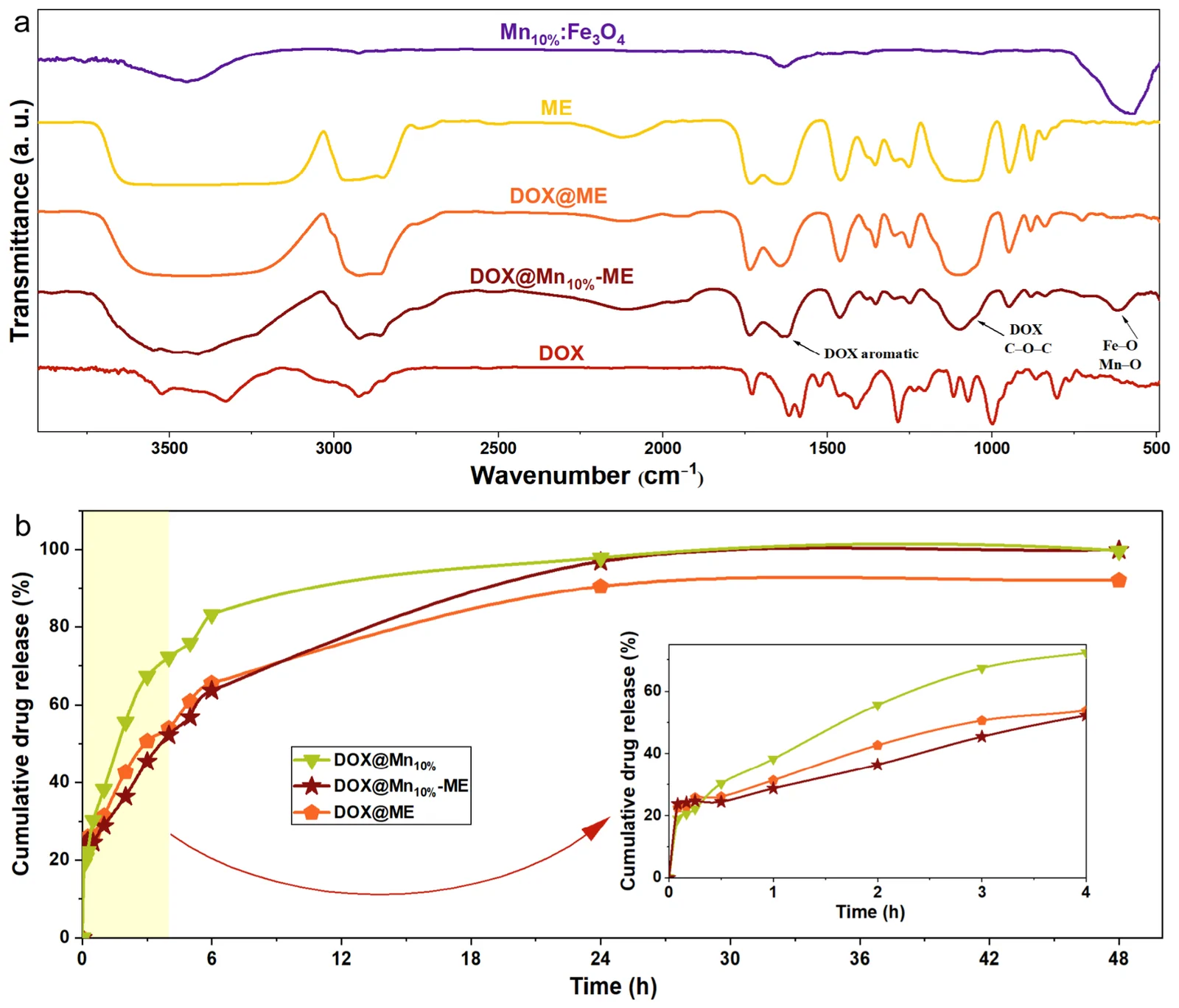
(**a**) FT-IR spectra of Mn_10%_:Fe_3_O_4_ nanoparticles, ME, DOX, and the corresponding drug-loaded formulations (DOX@ME and DOX@Mn_10%_-ME). (**b**) In vitro cumulative drug-release profiles of DOX from DOX@Mn_10%_, DOX@ME, and DOX@Mn_10%_-ME over 48 h; the inset highlights the initial release phase during the first 4 h (*n* = 2).

To gain insight into molecular interactions, FT-IR spectroscopy was employed (Figure 5a). The spectra of Mn_10%_ MNPs, ME, DOX, and the corresponding drug-loaded formulations (DOX@ME and DOX@Mn_10%_-ME) were compared to evaluate structural integrity and drug–matrix interactions.

The spectrum of pristine DOX exhibited the characteristic absorption bands of the anthracycline framework, including a broad O–H/N–H stretching band in the 3300–3400 cm^−1^ region, a prominent band at ~1610–1620 cm^−1^ corresponding to conjugated quinone/aromatic C=O/C=C vibrations, and intense signals in the 1200–1000 cm^−1^ region assigned to C–O and C–O–C stretching of the glycosidic moiety. These bands are consistent with the reported [63,64] vibrational features of DOX and confirm the preservation of its structural functionality. The unloaded ME displayed the typical spectral features of a Maisine CC/Tween 20-based system. Strong aliphatic C–H stretching bands were observed at ~2920 and ~2850 cm^−1^, while the ester carbonyl (C=O) vibration of the lipid phase appeared at ~1735 cm^−1^. The region around ~1100 cm^−1^ was dominated by C–O–C stretching vibrations associated with polyoxyethylene chains of Tween 20 and the ester backbone of the oil phase. The broad O–H stretching band reflects the presence of interfacial water and hydrogen-bonded surfactant groups. These findings confirm the structural integrity of the ME matrix [25,65,66]. Upon incorporation of DOX (DOX@ME), the spectrum retained the characteristic bands of the carrier system, whereas additional characteristic bands assigned to DOX became evident. In particular, the aromatic/quinone band (~1600 cm^−1^) became more pronounced compared with the unloaded ME, and the fingerprint region (1200–1000 cm^−1^) exhibited increased band complexity and intensity, indicating drug presence within the formulation. Moreover, a slight broadening of the O–H stretching region was observed, suggesting hydrogen-bonding interactions between DOX functional groups and the polar interfacial domains of ME. Importantly, the spectra did not reveal any new absorption bands indicative of covalent bond formation, suggesting that drug incorporation occurs primarily through non-covalent interactions.

In the final magnetic formulation (DOX@Mn_10%_-ME), the simultaneous presence of the ferrite lattice vibration (~570 cm^−1^), the ester carbonyl stretching vibration of the ME (~1735 cm^−1^), and characteristic vibrational bands associated with DOX (~1600 cm^−1^ and 1200–1000 cm^−1^) confirms the coexistence of all components within the formulation while preserving their characteristic vibrational signatures [63,67]. Notably, a slight shift and broadening of the quinone/aromatic band were observed relative to free DOX, which may reflect changes in the drug local chemical environment within the formulation. Such spectral changes could arise from interactions between DOX oxygen-containing functional groups and the MNPs’ surface, as well as hydrogen bonding within the interfacial region [32,68].

Collectively, the FT-IR data confirm (i) preservation of the structural integrity of DOX after encapsulation, (ii) maintenance of the ME architecture upon MNP incorporation, and (iii) successful hierarchical loading of DOX within the ME-coated Mn_10%_ system without evidence of chemical degradation. The MRI contrast capability of the developed system was quantitatively assessed through measurement of the longitudinal (*r*_1_) and transverse (*r*_2_) relaxivities. Relaxivity values represent the efficiency with which a contrast agent accelerates proton relaxation rates (R_1_ and R_2_), and therefore directly determine its MRI contrast performance [18].

In the context of the compositional screening discussed above, the Mn-doped series (Mn_1%_, Mn_5%_, and Mn_10%_:Fe_3_O_4_) exhibits a composition-dependent relaxometric response that does not follow a monotonic trend, in agreement with the non-linear evolution observed for magnetic parameters (Table 5). The Mn_1%_ sample shows the highest transverse relaxivity (*r*_2_ = 231.94 mM^−1^·s^−1^), indicating that low-level Mn incorporation is sufficient to generate strong local magnetic field inhomogeneities. Increasing the Mn content to 5% leads to a significant reduction in transverse relaxivity (*r*_2_ = 118.15 mM^−1^·s^−1^) while at 10%, Mn a moderate increase is observed (*r*_2_ = 133.01 mM^−1^·s^−1^), indicating a non-monotonic evolution of relaxometric performance across the series. This behavior suggests that Mn incorporation modulates the magnetic structure through competing effects on cation distribution and magnetic interactions, instead of simply enhancing the relaxometric performance. In this context, although Mn_1%_ provides the highest intrinsic *r*_2_, the Mn_10%_ composition retains strong *T*_2_ contrast capability while simultaneously exhibiting superior magnetic heating efficiency. This finding supports its selection as the optimal formulation for integration into the ME system. Such behavior is characteristic of superparamagnetic iron oxide-based nanoparticles acting as *T*_2_-weighted contrast agents, where relaxation is dominated by local magnetic field inhomogeneities generated by the ferrite cores. The high *r*_2_*/r*_1_ ratio therefore confirms that the Mn-doped ferrite behaves as a *T*_2_ contrast agent, consistent with previous reports showing that the relaxometric response of iron oxide-based nanoparticles strongly depends on parameters such as core composition, size, surface characteristics, hydrodynamic diameter, and aggregation state [18,69,70].

The blank ME showed very low relaxometric activity (*r*_1_ = 0.82 mM^−1^·s^−1^; *r*_2_ = 2.37 mM^−1^·s^−1^), indicating that the carrier matrix itself does not contribute significantly to proton relaxation. After the incorporation of Mn_10%_ into ME, the formulation exhibited a pronounced increase in relaxometric efficiency, reaching *r*_1_ = 6.15 mM^−1^·s^−1^ and *r*_2_ = 843.97 mM^−1^·s^−1^, with an *r*_2_/*r*_1_ ratio > 130, clearly revealing the *T*_2_-contrast agent capability of the MNP upon incorporation into ME. Because the measurements were performed at the same Fe concentration, this enhancement reflects a genuine modification of the magnetic relaxation environment. The increase can be associated with nanoparticle confinement and partial clustering within the ME structure, which strengthens dipolar interactions and generates larger magnetic field gradients experienced by diffusing water protons. Such an effect is consistent with the known dependence of *T*_2_ relaxivity on effective magnetic domain size and spatial organization, where controlled clustering or the confinement of superparamagnetic nanoparticles enhances transverse relaxation by amplifying susceptibility-induced magnetic heterogeneity. In this context, the ME acts not only as a carrier but also as a structuring medium that promotes a magnetic configuration more favorable for susceptibility-driven contrast enhancement [69,71].

The increase of *r*_1_ from 1.99 to 6.15 mM^−1^·s^−1^ remains significant. Although superparamagnetic ferrites mainly enhance transverse relaxation, longitudinal relaxivity can increase when water molecules interact more efficiently with the nanoparticle surface or remain longer in the magnetic field’s perturbed region. This suggests that the incorporation into ME modifies the local environment around them, affecting both interparticle magnetic interactions and water dynamics at the interface.

In addition, the observed increase in relaxivity can be further explained by proton exchange processes involving hydroxyl groups originating both from the Pluronic coating used during nanoparticle synthesis and from the ME components (Maisine CC, Tween 20, and ethanol). These systems provide a high density of exchangeable protons associated with –OH groups and interfacial water molecules located in close proximity to the magnetic cores. According to MRI relaxation principles, the efficiency of contrast agents strongly depends on the interaction between water protons and the local magnetic field generated by the nanoparticle surface, as well as on the distance and residence time of these protons near magnetic centers [72]. Under these conditions, proton exchange between interfacial hydroxyl groups and bulk water allows a larger fraction of protons to experience the magnetic perturbations, leading to more efficient relaxation pathways. This mechanism is conceptually consistent with chemical exchange-based contrast processes, where protons from the –OH groups can transfer magnetization to bulk water, thereby amplifying the MRI signal [73,74]. A similar contribution of hydroxyl-rich environments to enhanced relaxometric performance was also observed in our previous study [31] on Pluronic-stabilized ferrite systems, where increased proton accessibility and exchange dynamics were identified as key factors governing relaxation efficiency. Therefore, the overall enhancement in relaxivity arises from a synergistic effect between magnetic clustering and the presence of exchangeable protons at the nanoparticle–ME interface. In addition, Mn doping can influence the magnetic properties of the spinel lattice by modifying cation distribution and exchange interactions, which may further contribute to the observed relaxometric response [18,70,75].

When compared with clinically approved iron oxide MRI agents, the Mn_10%_ nanoparticle suspension exhibits transverse relaxivity values, obtained on a 1 T apparatus, within the range reported for several SPION formulations, including Ferumoxtran (*r*_2_ ≈ 65 mM^−1^·s^−1^), Ferucarbotran/Resovist (*r*_2_ ≈ 189 mM^−1^·s^−1^), Ferumoxide/Feridex (*r*_2_ ≈ 120 mM^−1^·s^−1^), Ferumoxytol (*r*_2_ ≈ 89 mM^−1^·s^−1^), and Ferumoxsil (*r*_2_ ≈ 47 mM^−1^·s^−1^), as reported under standardized conditions at 1.5 T, 37 °C, in water or plasma. Within this context, the measured *r*_2_ = 133.01 mM^−1^·s^−1^ for the Mn_10%_ nanoparticles are comparable to several clinically used *T*_2_ agents.

Notably, the Mn_10%_-ME formulation exhibits a markedly higher transverse relaxivity (*r*_2_ = 843.97 mM^−1^·s^−1^) obtained on the 1 T equipment, indicating that incorporation into the ME significantly enhances the susceptibility-driven relaxation efficiency of the magnetic phase. Although relaxivity values are influenced by experimental parameters, such as magnetic field strength, temperature, and dispersion medium, the magnitude of the observed *r*_2_ suggests a substantially improved *T*_2_ relaxometric performance compared with conventional SPION systems reported under standard conditions. These results therefore highlight the ability of the ME-based architecture to amplify the magnetic relaxation response, yielding a system with superior transverse contrast efficiency under the present measurement conditions.

Beyond improving imaging performance, the ME structure also provides a functional environment for the incorporation and controlled release of therapeutic agents. Therefore, the following section examines the DOX-release behavior from the DOX@Mn_10%_-ME system.

Controlled release experiments performed using the dialysis method in a phosphate buffer at 37 °C under sink conditions and at constant volume revealed that all formulations follow a biphasic release profile, characterized by an initial burst phase followed by sustained release (Figure 5b). The DOX@Mn_10%_ nanoparticles exhibited the fastest release kinetics, reaching high cumulative release within the early time window and approaching complete release after 48 h. This behavior is consistent with the passive adsorption mechanism used for drug loading, where a significant fraction of DOX is located at or near the nanoparticle surface and is therefore readily exposed to the surrounding medium, resulting in rapid initial desorption and diffusion into the surrounding medium [76].

The DOX-loaded ME without nanoparticles (DOX@ME) displayed a slower and more gradual release profile. In this system, the drug is expected to partition within the oil phase and interfacial regions of droplets. Its release requires transfer into the external aqueous phase prior to diffusion through the dialysis membrane. Such release from ME-based systems is governed by the distribution of drug molecules among oil, aqueous, and interfacial domains. The transfer from the oil phase to the aqueous phase represents an additional mass-transfer step that slows the overall release rate. Therefore, the slower release behavior can be attributed to this partitioning process between the internal droplet domains and the external aqueous phase, which introduces a diffusional resistance and moderates the initial burst [77,78].

The DOX@Mn_10%_-ME formulation exhibits an intermediate yet advantageous release profile between those observed for DOX@Mn_10%_ and DOX@ME. The pronounced initial burst seen for DOX@Mn_10%_ alone is significantly attenuated, indicating that the ME matrix introduces an additional diffusional barrier around the adsorbed drug. Under these conditions, drug release proceeds through a combined mechanism involving desorption from the nanoparticle surface followed by diffusion through a structured ME interfacial network. The structured organization of the surfactant-oil domains, therefore, slows drug transport while maintaining drug accessibility. Importantly, near-complete cumulative release is still achieved after 48 h, indicating that ME effectively controls release kinetics without permanently trapping the drug.

Taken together, these results indicate that ME architecture simultaneously influences magnetic and transport properties. The same microstructural confinement that enhances MRI relaxivity and maintains high magnetic performance also modulates drug diffusion, reducing burst release while enabling sustained delivery. Thus, the ME matrix does not act merely as a carrier but as a functional environment that integrates imaging capability with controlled chemotherapeutic release, supporting the development of a multifunctional nanoplatform with intended theranostic applications.

As demonstrated throughout this study, the integration of dopant-tuned MNPs within the ME matrix enables a coordinated modulation of magnetic heating efficiency, MRI relaxometric response, and chemotherapeutic release kinetics. The dual functionality of the magnetic core composition and carrier architecture highlights the versatility of the proposed system, which is capable of simultaneously integrating imaging capability, hyperthermia, and controlled drug delivery.

## 4. Conclusions

Mn-doped MNPs with 1%, 5%, and 10% Mn and an unaltered Fe^2+^/Fe^3+^ ratio were successfully synthesized and incorporated into a previously optimized oil-in-water ME platform to obtain a multifunctional system combining magnetic functionality, MRI contrast performance, and controlled drug delivery. Structural characterization confirmed the preservation of the cubic spinel phase across the investigated doping range, with progressive lattice expansion indicating the incorporation of Mn into a ferrite lattice without the formation of secondary phases. The nanoparticles remained within the nanometric crystallite size range (~9–12 nm) and exhibited superparamagnetic behavior at room temperature. Magnetic measurements demonstrated that Mn incorporation modulates the magnetic properties of magnetite while maintaining low remanence and coercivity in line with requirements for medical applications. Among investigated compositions, the Mn_10%_ sample displayed the highest heating efficiency under alternating magnetic fields, indicating that the compositional tuning of ferrite lattice can enhance hyperthermia performance, with SAR values increasing from 70 to 111 W·g^−1^ as Mn content in the nanoparticles increases. Relaxometric analysis further showed that Mn doping confers strong transverse relaxation properties characteristic of *T*_2_ contrast agents, with values similar to clinically approved Fe-based contrast agents. Notably, the incorporation of the nanoparticles into the ME matrix resulted in a pronounced increase in transverse relaxivity, consistent with nanoparticle confinement and enhanced magnetic susceptibility effects within the structured carrier environment. This can also be attributed to the presence of the hydroxyl-rich interfacial regions of ME’s components, which may facilitate proton exchange processes and increase the fraction of water protons interacting with the local magnetic field. The integration of Mn_10%_:Fe_3_O_4_ and DOX into the ME system preserved the oil-in-water architecture while enabling simultaneous magnetic functionality and drug loading. Release experiments revealed that the ME matrix moderates the rapid desorption observed for nanoparticle-bound DOX, leading to a more controlled release profile compared with the nanoparticle suspension alone. Equally important, the ME-based nanoformulation containing the DOX loaded on Mn_10%_:Fe_3_O_4_ maintained colloidal stability after six months of storage at 4 °C. Collectively, these results demonstrate that the combination of dopant-engineered MNPs with a thermodynamically stable ME carrier enables the simultaneous integration of magnetic hyperthermia potential, MRI contrast capability, and controlled drug release. The proposed architecture therefore represents a promising platform for the development of a multifunctional nanoplatform with potential theranostic applications based on magnetically responsive nanocarriers. Future studies will focus on in vitro biological evaluation to assess cellular uptake, cytocompatibility, and therapeutic efficacy—critical steps for validating the translational potential of the nanoparticles.

## Figures and Tables

**Table 2:** ICP-OES (*n* = 3), DLS (*n* = 3), and ζ-potential (*n* = 5) results for all the synthesized samples.

Sample	Nominal Composition		ICP-OES		DLS		
	Fe (mg·mL^−1^)	Mn (mg·mL^−1^)	Fe (mg·mL^−1^)	Mn (mg·mL^−1^)	Hydrodynamic Size (nm)	PDI	ζ-Potential (mV)
**Mn_1%_**	4.43	0.074	4.214 ± 0.039	0.069 ± 0.001	212 ± 1.688	0.200 ± 0.011	+2.56 ± 0.522
**Mn_5%_**	4.43	0.343	3.974 ± 0.074	0.304 ± 0.004	214 ± 4.658	0.193 ± 0.025	+7.68 ± 0.712
**Mn_10%_**	4.43	0.685	3.858 ± 0.047	0.586 ± 0.001	316 ± 3.705	0.193 ± 0.087	+20.36 ± 0.991

Note: Replicates represent repeated measurements of the same sample.

**Table 3:** Values of the hysteresis parameters for Mn low-level doped Fe_3_O_4_.

Sample	M_s_ (emu/g)	M_r_ (emu/g)	H_c_ (Oe)	M_r_/M_s_
**Mn_1%_**	49.23	1.01	12.41	0.022
**Mn_5%_**	59.21	1.83	17.37	0.031
**Mn_10%_**	55.1	2.41	23.25	0.044

**Table 5:** Longitudinal (*r*_1_) and transverse (*r*_2_) relaxivities, together with the *r*_2_/*r*_1_ ratio, determined for pristine ME, Mn_1, 5, 10%_:Fe_3_O_4_, and the nanoparticle-loaded ME (Mn_10%_-ME).

Sample	*r*_1_ [mM^−1^·s^−1^]	*r*_2_ [mM^−1^·s^−1^]	*r*_2_/*r*_1_
**ME**	0.82	2.37	2.89
**Mn_1%_:Fe_3_O_4_**	3.37	231.94	68.82
**Mn_5%_:Fe_3_O_4_**	1.50	118.15	78.76
**Mn_10%_:Fe_3_O_4_**	1.99	133.01	66.83
**Mn_10%_-ME**	6.15	843.97	137.23

## Data Availability

The original contributions presented in this study are included in the article/Supplementary Material. Further inquiries can be directed to the corresponding authors.

## Supplementary Materials

The following supporting information can be downloaded at: https://www.mdpi.com/article/10.3390/nano16171065/s1, Figure S1: Cumulative DOX release profiles of (a) DOX@Mn_10%_, (b) DOX@ME, and (c) DOX@Mn_10%_-ME over 2880 min (48h). Duplicate measurements are included for each formulation.; Figure S2: Calibration curve of doxorubicin in PB (pH 7.4).; Table S1: Electrical conductivity, hydrodynamic diameter (D_h_, n = 3), polydispersity index (PDI, n = 3), and ζ-potential values (n = 5) of MNPs synthesised samples.; Table S2: Electrical conductivity, hydrodynamic diameter (D_h_, n = 3), polydispersity index (PDI, n = 3), and ζ-potential values (n = 6).

## References

[1] Mitra D. (2023). Microemulsion and its application: An inside story. Mater. Today Proc..

[2] Yadav K.S., Soni G., Choudhary D., Khanduri A., Bhandari A., Joshi G. (2023). Microemulsions for enhancing drug delivery of hydrophilic drugs: Exploring various routes of administration. Med. Drug Discov..

[3] Jadhav K.R., Shaikh I.M., Ambade K.W., Kadam V.J. (2006). Applications of microemulsion based drug delivery system. Curr. Drug Deliv..

[4] Suhail N., Alzahrani A.K., Basha W.J., Kizilbash N., Zaidi A., Ambreen J., Khachfe H.M. (2021). Microemulsions: Unique Properties, Pharmacological Applications, and Targeted Drug Delivery. Front. Nanotechnol..

[5] Linders A.N., Dias I.B., López Fernández T., Tocchetti C.G., Bomer N., Van der Meer P. (2024). A review of the pathophysiological mechanisms of doxorubicin-induced cardiotoxicity and aging. npj Aging.

[6] Kciuk M., Gielecińska A., Mujwar S., Kołat D., Kałuzińska-Kołat Ż., Celik I., Kontek R. (2023). Doxorubicin—An Agent with Multiple Mechanisms of Anticancer Activity. Cells.

[7] Sritharan S., Sivalingam N. (2021). A comprehensive review on time-tested anticancer drug doxorubicin. Life Sci..

[8] Gavilán H., Avugadda S.K., Fernández-Cabada T., Soni N., Cassani M., Mai B.T., Chantrell R., Pellegrino T. (2021). Magnetic nanoparticles and clusters for magnetic hyperthermia: Optimizing their heat performance and developing combinatorial therapies to tackle cancer. Chem. Soc. Rev..

[9] Li Z., Bai R., Yi J., Zhou H., Xian J., Chen C. (2023). Designing Smart Iron Oxide Nanoparticles for MR Imaging of Tumors. Chem. Biomed. Imaging.

[10] Materón E.M., Miyazaki C.M., Carr O., Joshi N., Picciani P.H.S., Dalmaschio C.J., Davis F., Shimizu F.M. (2021). Magnetic nanoparticles in biomedical applications: A review. Appl. Surf. Sci. Adv..

[11] Anik M.I., Hossain M.K., Hossain I., Mahfuz A.M.U.B., Rahman M.T., Ahmed I. (2021). Recent progress of magnetic nanoparticles in biomedical applications: A review. Nano Sel..

[12] Liu X., Zhang Y., Wang Y., Zhu W., Li G., Ma X., Zhang Y., Chen S., Tiwari S., Shi K. (2020). Comprehensive understanding of magnetic hyperthermia for improving antitumor therapeutic efficacy. Theranostics.

[13] Bañobre-López M., Teijeiro A., Rivas J. (2013). Magnetic nanoparticle-based hyperthermia for cancer treatment. Rep. Pract. Oncol. Radiother..

[14] Xiong L., Liang B., Yu K. (2025). Magnetic hyperthermia in oncology: Nanomaterials-driven combinatorial strategies for synergistic therapeutic gains. Mater. Today Bio.

[15] Rosensweig R.E. (2002). Heating magnetic fluid with alternating magnetic field. J. Magn. Magn. Mater..

[16] Hergt R., Dutz S. (2007). Magnetic particle hyperthermia—Biophysical limitations of a visionary tumour therapy. J. Magn. Magn. Mater..

[17] Jeon M., Halbert M.V., Stephen Z.R., Zhang M. (2021). Iron Oxide Nanoparticles as T1 Contrast Agents for Magnetic Resonance Imaging: Fundamentals, Challenges, Applications, and Prospectives. Adv. Mater..

[18] Aboushoushah S.F.O. (2025). Iron oxide nanoparticles enhancing magnetic resonance imaging: A review of the latest advancements. J. Sci. Adv. Mater. Devices.

[19] Lapusan R., Borlan R., Focsan M. (2024). Advancing MRI with magnetic nanoparticles: A comprehensive review of translational research and clinical trials. Nanoscale Adv..

[20] Shubayev V.I., Pisanic T.R., Jin S. (2009). Magnetic nanoparticles for theragnostics. Adv. Drug Deliv. Rev..

[21] Rodríguez C.F., Guzmán-Sastoque P., Santacruz-Belalcazar A., Rodriguez C., Villamarin P., Reyes L.H., Cruz J.C. (2025). Magnetoliposomes for nanomedicine: Synthesis, characterization, and applications in drug, gene, and peptide delivery. Expert Opin. Drug Deliv..

[22] Kneidl B., Peller M., Winter G., Lindner L.H., Hossann M. (2014). Thermosensitive liposomal drug delivery systems: State of the art review. Int. J. Nanomed..

[23] Tomitaka A., Takemura Y., Huang Z., Roy U., Nair M. (2019). Magnetoliposomes in Controlled-Release Drug Delivery Systems. Crit. Rev. Biomed. Eng..

[24] An Y., Yang R., Wang X., Han Y., Jia G., Hu C., Zhang Z., Liu D., Tang Q. (2021). Facile Assembly of Thermosensitive Liposomes for Active Targeting Imaging and Synergetic Chemo-/Magnetic Hyperthermia Therapy. Front. Bioeng. Biotechnol..

[25] Nistor M., Nicolescu A., Amarandi R.-M., Pui A., Stiufiuc R.-I., Dragoi B. (2025). Multi spectroscopic investigation of maisine-based microemulsions as convenient carriers for co-delivery of anticancer and anti-inflammatory drugs. Sci. Rep..

[26] Hosny A.A., El-Deeb B., Mohamed Z.A., Hassan A., Kamel M., Elbeltagi S., Saadallah H.A.A., Ibrahim E.M.M. (2025). Green synthesis of Mn-doped iron oxide nanoparticles using sugarcane juice for magnetic hyperthermia applications. Sci. Rep..

[27] Gupta R., Tomar R., Chakraverty S., Sharma D. (2021). Effect of manganese doping on the hyperthermic profile of ferrite nanoparticles using response surface methodology. RSC Adv..

[28] Odio O.F., Tommasini G., Teran F.J., Ovejero J.G., Rubín J., Moros M., Del Sol-Fernández S. (2025). Unraveling the Mn2+ substitution effect on the anisotropy control and magnetic hyperthermia of MnxFe3−xO4 nanoparticles. Nanoscale Horiz..

[29] Lee J.H., Huh Y.M., Jun Y.W., Seo J.W., Jang J.T., Song H.T., Kim S., Cho E.J., Yoon H.G., Suh J.S. (2007). Artificially engineered magnetic nanoparticles for ultra-sensitive molecular imaging. Nat. Med..

[30] Zhen Z., Xie J. (2012). Development of manganese-based nanoparticles as contrast probes for magnetic resonance imaging. Theranostics.

[31] Nistor M., Balan V., Pui A., Zara-Danceanu C.M., Postu P.A., Grigoras M., Gherca D., Toma V., Uritu C.M., Fratila R.M. (2026). Precise-engineering of monodisperse magnetite nanoparticles with low-level of cobalt doping for enhanced MRI contrast performance. Mater. Des..

[32] Pasieczna-Patkowska S., Cichy M., Flieger J. (2025). Application of Fourier Transform Infrared (FTIR) Spectroscopy in Characterization of Green Synthesized Nanoparticles. Molecules.

[33] Branca C., Khouzami K., Wanderlingh U., D’Angelo G. (2018). Effect of intercalated chitosan/clay nanostructures on concentrated pluronic F127 solution: A FTIR-ATR, DSC and rheological study. J. Colloid Interface Sci..

[34] Lin J.-J., Chen J.-S., Huang S.-J., Ko J.-H., Wang Y.-M., Chen T.-L., Wang L.-F. (2009). Folic acid–Pluronic F127 magnetic nanoparticle clusters for combined targeting, diagnosis, and therapy applications. Biomaterials.

[35] Mary Jacintha A., Umapathy V., Neeraja P., Rex Jeya Rajkumar S. (2017). Synthesis and comparative studies of MnFe2O4 nanoparticles with different natural polymers by sol–gel method: Structural, morphological, optical, magnetic, catalytic and biological activities. J. Nanostruct. Chem..

[36] Sureshkumar V., Kiruba Daniel S.C.G., Ruckmani K., Sivakumar M. (2016). Fabrication of chitosan–magnetite nanocomposite strip for chromium removal. Appl. Nanosci..

[37] Bhattacharjee S. (2016). DLS and zeta potential—What they are and what they are not?. J. Control. Release.

[38] Stetefeld J., McKenna S.A., Patel T.R. (2016). Dynamic light scattering: A practical guide and applications in biomedical sciences. Biophys. Rev..

[39] Laurent S., Forge D., Port M., Roch A., Robic C., Vander Elst L., Muller R.N. (2008). Magnetic Iron Oxide Nanoparticles: Synthesis, Stabilization, Vectorization, Physicochemical Characterizations, and Biological Applications. Chem. Rev..

[40] Joudeh N., Linke D. (2022). Nanoparticle classification, physicochemical properties, characterization, and applications: A comprehensive review for biologists. J. Nanobiotechnol..

[41] Wu W., Wu Z., Yu T., Jiang C., Kim W.-S. (2015). Recent progress on magnetic iron oxide nanoparticles: Synthesis, surface functional strategies and biomedical applications. Sci. Technol. Adv. Mater..

[42] Yan Z., Chaluvadi A., FitzGerald S., Spence S., Bleyer C., Zhu J., Crawford T.M., Getman R.B., Watt J., Huber D.L. (2022). Effect of manganese substitution of ferrite nanoparticles on particle grain structure. Nanoscale Adv..

[43] Shebha Anandhi J., Antilen Jacob G., Justin Joseyphus R. (2020). Factors affecting the heating efficiency of Mn-doped Fe3O4 nanoparticles. J. Magn. Magn. Mater..

[44] Garg P., Krishna M., Horne D., Salgia R., Singhal S.S. (2025). Hyperthermia-induced apoptosis: The role of magnetic nanoparticles in targeted delivery for breast cancer treatment. Biochim. Biophys. Acta (BBA) Rev. Cancer.

[45] Roy P., Ganguly R., Biswas N. (2025). A critical review on magnetic fluid hyperthermia treatment for cancers: Challenges and pathways from computation to clinic. J. Therm. Biol..

[46] Iacovita C., Florea A., Scorus L., Pall E., Dudric R., Moldovan A.I., Stiufiuc R., Tetean R., Lucaciu C.M. (2019). Hyperthermia, Cytotoxicity, and Cellular Uptake Properties of Manganese and Zinc Ferrite Magnetic Nanoparticles Synthesized by a Polyol-Mediated Process. Nanomaterials.

[47] Vega-Carrasco E.R., Ansari S.R., Zhao J., del Carmen Suárez-López Y., Larsson P., Teleki A. (2026). Machine Learning on Systematically Curated Data Reveals Key Determinants of Magnetic Hyperthermia Performance. Small.

[48] Callender S.P., Mathews J.A., Kobernyk K., Wettig S.D. (2017). Microemulsion utility in pharmaceuticals: Implications for multi-drug delivery. Int. J. Pharm..

[49] Acharya D.P., Hartley P.G. (2012). Progress in microemulsion characterization. Curr. Opin. Colloid Interface Sci..

[50] Lawrence M.J., Rees G.D. (2012). Microemulsion-based media as novel drug delivery systems. Adv. Drug Deliv. Rev..

[51] Eastoe J., Hatzopoulos M.H., Tabor R., Tadros T. (2013). Microemulsions. Encyclopedia of Colloid and Interface Science.

[52] Musakhanian J., Osborne D.W. (2025). Understanding Microemulsions and Nanoemulsions in (Trans)Dermal Delivery. AAPS PharmSciTech.

[53] Esen Yigit Ö., Lamprecht A. (2026). Impact of Drug Hydrophilicity on Transdermal Delivery by Nanoemulsions. Pharmaceutics.

[54] Egito E.S.T., Amaral-Machado L., Alencar E.N., Oliveira A.G. (2021). Microemulsion systems: From the design and architecture to the building of a new delivery system for multiple-route drug delivery. Drug Deliv. Transl. Res..

[55] Pellosi D.S., Macaroff P.P., Morais P.C., Tedesco A.C. (2018). Magneto low-density nanoemulsion (MLDE): A potential vehicle for combined hyperthermia and photodynamic therapy to treat cancer selectively. Mater. Sci. Eng. C.

[56] Kichatov B., Korshunov A., Sudakov V., Petrov O., Gubernov V., Korshunova E., Kolobov A., Kiverin A. (2022). Magnetic Nanomotors in Emulsions for Locomotion of Microdroplets. ACS Appl. Mater. Interfaces.

[57] Takechi-Haraya Y., Ohgita T., Demizu Y., Saito H., Izutsu K.-i., Sakai-Kato K. (2022). Current Status and Challenges of Analytical Methods for Evaluation of Size and Surface Modification of Nanoparticle-Based Drug Formulations. AAPS PharmSciTech.

[58] Sarkar B., Venugopal V., Bodratti A.M., Tsianou M., Alexandridis P. (2013). Nanoparticle surface modification by amphiphilic polymers in aqueous media: Role of polar organic solvents. J. Colloid Interface Sci..

[59] Kabong M.A., Focke W.W., Du Toit E.L., Rolfes H., Ramjee S. (2020). Breakdown mechanisms of oil-in-water emulsions stabilised with Pluronic F127 and co-surfactants. Colloids Surf. A Physicochem. Eng. Asp..

[60] Akash M.S.H., Rehman K. (2015). Recent progress in biomedical applications of Pluronic (PF127): Pharmaceutical perspectives. J. Control. Release.

[61] Gonzales M., Krishnan K.M. (2007). Phase transfer of highly monodisperse iron oxide nanocrystals with Pluronic F127 for biomedical applications. J. Magn. Magn. Mater..

[62] Harman C.L.G., Patel M.A., Guldin S., Davies G.-L. (2019). Recent developments in Pickering emulsions for biomedical applications. Curr. Opin. Colloid Interface Sci..

[63] Bansal R., Singh R., Kaur K. (2021). Quantitative analysis of doxorubicin hydrochloride and arterolane maleate by mid IR spectroscopy using transmission and reflectance modes. BMC Chem..

[64] Szafraniec E., Majzner K., Farhane Z., Byrne H.J., Lukawska M., Oszczapowicz I., Chlopicki S., Baranska M. (2016). Spectroscopic studies of anthracyclines: Structural characterization and in vitro tracking. Spectrochim. Acta Part A Mol. Biomol. Spectrosc..

[65] Lewis R.N., McElhaney R.N. (2007). Fourier transform infrared spectroscopy in the study of lipid phase transitions in model and biological membranes: Practical considerations. Methods Mol. Biol..

[66] Yusuf H.H., Roddick F., Pramanik B.K. (2026). Sodium alginate—Tween 20 dispersant for sustainable in-sewer control of fats, oils, and grease (FOG) accumulation. Water Res..

[67] Das J., Choi Y.-J., Han J.W., Reza A.M.M.T., Kim J.-H. (2017). Nanoceria-mediated delivery of doxorubicin enhances the anti-tumour efficiency in ovarian cancer cells via apoptosis. Sci. Rep..

[68] Kayal S., Ramanujan R.V. (2010). Doxorubicin loaded PVA coated iron oxide nanoparticles for targeted drug delivery. Mater. Sci. Eng. C.

[69] Dadfar S.M., Roemhild K., Drude N.I., von Stillfried S., Knüchel R., Kiessling F., Lammers T. (2019). Iron oxide nanoparticles: Diagnostic, therapeutic and theranostic applications. Adv. Drug Deliv. Rev..

[70] Ghazi R., Ibrahim T.K., Nasir J.A., Gai S., Ali G., Boukhris I., Rehman Z. (2025). Iron oxide based magnetic nanoparticles for hyperthermia, MRI and drug delivery applications: A review. RSC Adv..

[71] Yang Y., Liu Y., Song L., Cui X., Zhou J., Jin G., Boccaccini A.R., Virtanen S. (2023). Iron oxide nanoparticle-based nanocomposites in biomedical application. Trends Biotechnol..

[72] Basini M., Guerrini A., Cobianchi M., Orsini F., Bettega D., Avolio M., Innocenti C., Sangregorio C., Lascialfari A., Arosio P. (2019). Tailoring the magnetic core of organic-coated iron oxides nanoparticles to influence their contrast efficiency for Magnetic Resonance Imaging. J. Alloys Compd..

[73] Shaffer J.J., Mani M., Schmitz S.L., Xu J., Owusu N., Wu D., Magnotta V.A., Wemmie J.A. (2020). Proton Exchange Magnetic Resonance Imaging: Current and Future Applications in Psychiatric Research. Front. Psychiatry.

[74] van Zijl P.C., Yadav N.N. (2011). Chemical exchange saturation transfer (CEST): What is in a name and what isn’t?. Magn. Reson. Med..

[75] Okeke I.S., Echeweozo E.O., Ogu A.C., Aondona P.Y., Ezema F.I. (2024). Iron oxide Nanoparticles; A Review on the role of Transition Metal doping on its magnetic properties and particle size for imaging and therapeutic applications. Hybrid Adv..

[76] Palanikumar L., Al-Hosani S., Kalmouni M., Nguyen V.P., Ali L., Pasricha R., Barrera F.N., Magzoub M. (2020). pH-responsive high stability polymeric nanoparticles for targeted delivery of anticancer therapeutics. Commun. Biol..

[77] Dong Y., Hengst L., Hunt R., Patel D., Vo A., Choi S., Ashraf M., Cruz C.N., Xu X. (2019). Understanding drug distribution and release in ophthalmic emulsions through quantitative evaluation of formulation-associated variables. J. Control. Release.

[78] Modi S., Anderson B.D. (2013). Determination of Drug Release Kinetics from Nanoparticles: Overcoming Pitfalls of the Dynamic Dialysis Method. Mol. Pharm..

