# Supplementary material for: Design and Physicochemical Characterization of a Multifunctional Maisine-Based Microemulsion Incorporating Doxorubicin@Mn-Doped Magnetite Nanoparticles for MRI, Hyperthermia, and Drug Delivery
Source: Nanomaterials (Basel). 2026 Aug 26;16(17):1065. doi: 10.3390/nano16171065 (PMC13567371; doi:10.3390/nano16171065)
Supplement: Supplementary file 1 [file nanomaterials-16-01065-s001.zip › nanomaterials-4427019-supplementary.pdf]

Supplementary Information

Design and Physicochemical Characterization of a Multifunctional Maisine-Based Microemulsion Incorporating Doxorubicin@Mn-Doped Magnetite Nanoparticles for MRI, Hyperthermia, and Drug Delivery

Mirela Nistor [1,2], Daniel Gherca [3], Cristina Mariana Uritu [4], Marian Grigoras [3], Vera Balan [1,5], Raluca M. Fratila [6,7], Rares-Ionut Stiufiuc [1,8], Brindusa Dragoi [1,2,]* and Aurel Pui [2,]*

1 Nanotechnology Laboratory, TRANSCEND Department, Regional Institute of Oncology, 2-4 General Henri Mathias Berthelot, 700483 Iasi, Romania; mirela.nistor@chem.uaic.ro (M.N.); balan.vera@umfiasi.ro (V.B.); rares.stiufiuc@umfcluj.ro (R.-I.S.)
2 Faculty of Chemistry, "Alexandru Ioan Cuza" University of Iasi, 11 Carol I Blvd., 700506 Iasi, Romania
3 National Institute of Research and Development for Technical Physics, 700050 Iasi, Romania; dgherca@phys-iasi.ro (D.G.); mgrigoras@phys-iasi.ro (M.G.)
4 Advanced Centre for Research-Development in Experimental Medicine "Prof. Ostin C. Mungiu" (CEMEX), Grigore T. Popa University of Medicine and Pharmacy, 9-13 Mihail Kogalniceanu, 700259 Iasi, Romania; cristina-mariana.uritu@umfiasi.ro
5 Biomedical Sciences Department, Faculty of Medical Bioengineering, Grigore T. Popa University of Medicine and Pharmacy of Iasi, 9-13 Kogalniceanu Street, 700454 Iasi, Romania
6 Instituto de Nanociencia y Materiales de Aragón, INMA (CSIC-Universidad de Zaragoza), C/Pedro Cerbuna 12, 50009 Zaragoza, Spain; rfratila@unizar.es
7 Centro de Investigación Biomédica en Red de Bioingeniería, Biomateriales y Nanomedicina (CIBER-BBN), 50018 Zaragoza, Spain
8 Department of NanoSciences, Institute of Biomedical Research—MedFUTURE, "Iuliu Hatieganu" University of Medicine and Pharmacy, Louis Pasteur 4-6, 400349 Cluj Napoca, Romania
* Correspondence: brindusa.dragoi@yahoo.com (B.D.); aurel@uaic.ro (A.P.)

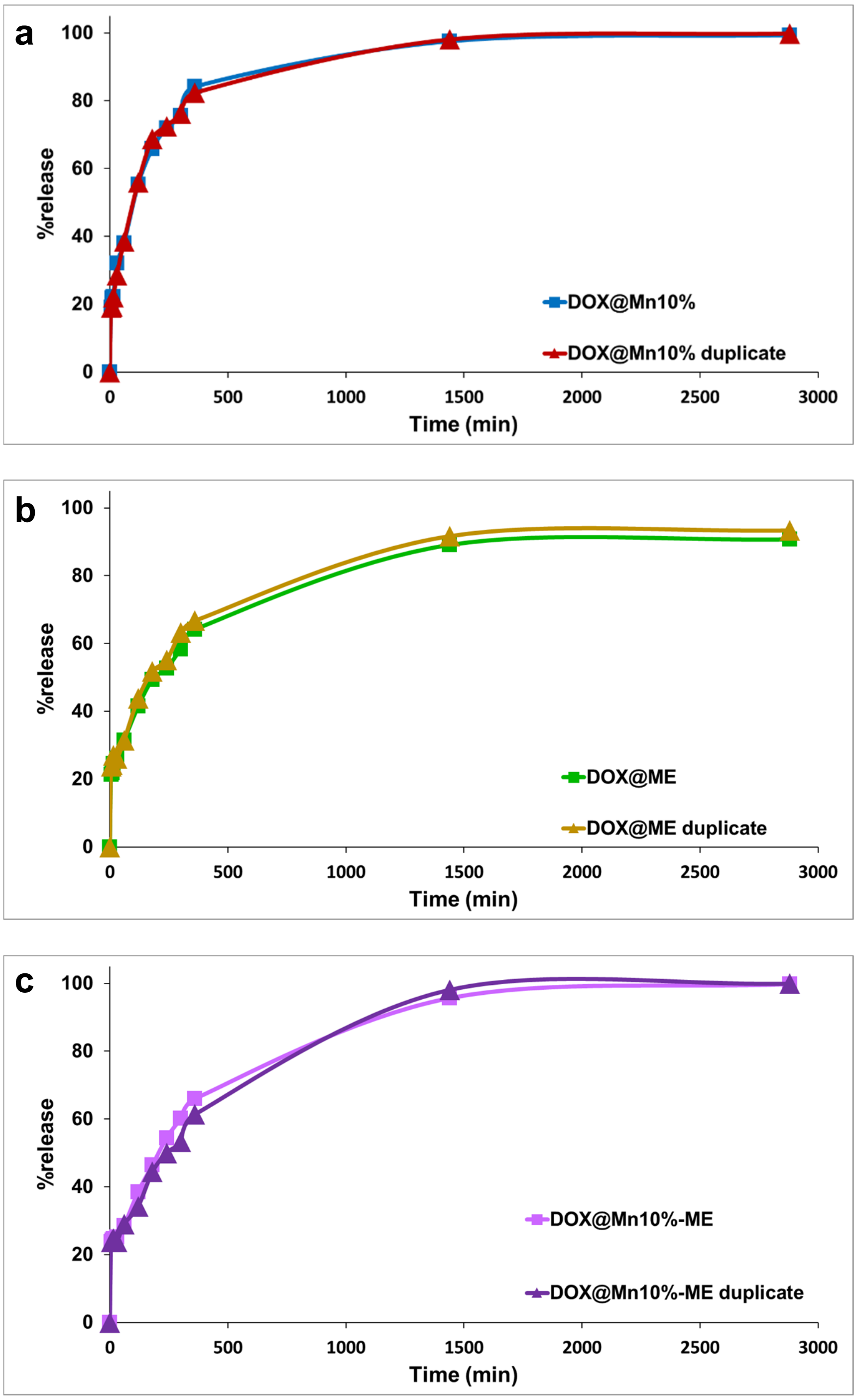

Figure S1. Cumulative DOX release profiles of (a) DOX@$Mn_{10\%}$, (b) DOX@ME, and (c) DOX@$Mn_{10\%}$-ME over 2880 min (48h). Duplicate measurements are included for each formulation.

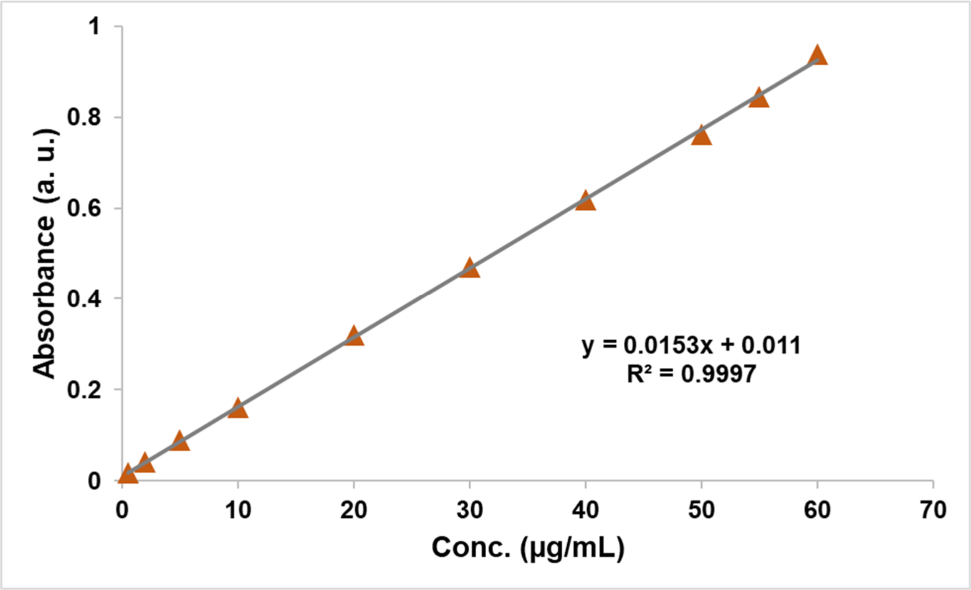

Figure S2. Calibration curve of doxorubicin in PB (pH 7.4).

Table S1. Electrical conductivity, hydrodynamic diameter (D_h, n = 3), polydispersity index (PDI, n = 3), and ζ-potential values (n = 5) of MNPs synthesised samples.

Sample	ICP-OES						DLS								
	Fe ($mg \cdot mL^{-1}$)	Mean	StDev	Mn ($mg \cdot mL^{-1}$)	Mean	StDev	D_h (nm)	Mean	StDev	PDI	Mean	StDev	ζ-potential (mV)	Mean	StDev
$Mn_{1\%}$	4.178			0.069			211.82			0.210			+2.5		
	4.210	4.214	0.039	0.068	0.069	0.001	211.02	212.37	1.688	0.203	0.200	0.011	+2.4		
	4.255			0.069			214.26			0.188			+3	+2.56	0.522
													+1.8		
													+3.1		
$Mn_{5\%}$	4.055			0.308			209.71			0.193			+7.5		
	3.910	3.974	0.074	0.301	0.304	0.004	218.95	213.99	4.658	0.168	0.193	0.025	+7		
	3.957			0.303			213.3			0.218			+7.9	+7.68	0.712
													+8.8		
													+7.2		
$Mn_{10\%}$	3.839			0.587			313.35			0.255	0.194	0.087	+20.6		
	3.912	3.858	0.047	0.586	0.586	0.001	314.25	315.92	3.705	0.132			+19		
	3.823			0.586			320.17						+21.3	+20.36	0.991
													+21.2		
													+19.7		

Note: Replicates represent repeated measurements of the same sample.

Table S2. Electrical conductivity, hydrodynamic diameter (D_h, n = 3), polydispersity index (PDI, n = 3), and ζ-potential values (n = 6).

Sample	Measurement	Date	DLS								
			D_h (nm)	Mean	StDev	PDI	Mean	StDev	ζ-potential(mV)	Mean	StDev
Freshly prepared											
**ME**	1	19.01.2026	168.78	170.41	1.733	0.3149	0.292	0.031	-21.2	-20.85	0.599
	2		170.21			0.3034			-20.8		
	3		172.23			0.2572			-21.7		
	4								-20.4		
	5								-21		
	6								-20		
**DOX@Mn$_{10\%}$-ME**	1	19.01.2026	276.35	274.33	3.092	0.3216	0.314	0.023	-16.5	-14.62	1.280
	2		270.77			0.3319			-12.7		
	3		275.87			0.2886			-14		
	4								-15		
	5								-15.2		
	6								-14.3		
**DOX@ME**	1	19.01.2026	126.05	126.41	1.469	0.2838	0.277	0.018	-17.7	-17.35	1.097
	2		125.16			0.2905			-15.6		
	3		128.03			0.2567			-17.2		
	4								-17.9		
	5								-17.4		
	6								-18.3		
After 6 months											
**ME**	1	20.07.2026	193.05	187.72	5.340	0.359	0.3642	0.008	-24	-22.95	0.660
	2		187.74			0.3735			-22.6		
	3		182.37			0.36			-23.5		
	4								-22.3		
	5								-22.5		
	6								-22.8		
**DOX@Mn$_{10\%}$-ME**	1	20.07.2026	276.77	281.36	4.836	0.3104	0.2989	0.024	-19	-17.52	1.250
	2		286.41			0.2712			-18.3		
	3		280.9			0.3152			-17.7		
	4								-18.1		
	5								-15.9		
	6								-16.1		
**DOX@ME**	1	20.07.2026	252.83	249.20	3.534	0.3795	0.3844	0.007	-24.7	-23.98	0.605
	2		249.01			0.3814			-24.4		
	3		245.77			0.3922			-23.2		
	4								-24.2		

5	-23.3
6	-24.1

Note: Replicates represent repeated measurements of the same sample.
